# Cuf2 Is a Transcriptional Co-Regulator that Interacts with Mei4 for Timely Expression of Middle-Phase Meiotic Genes

**DOI:** 10.1371/journal.pone.0151914

**Published:** 2016-03-17

**Authors:** Raphaël Ioannoni, Ariane Brault, Simon Labbé

**Affiliations:** Département de Biochimie, Faculté de médecine et des sciences de la santé, Université de Sherbrooke, Sherbrooke, QC, J1E 4K8, Canada; University of Cambridge, UNITED KINGDOM

## Abstract

The *Schizosaccharomyces pombe cuf2*^*+*^ gene encodes a nuclear regulator that is required for timely activation and repression of several middle-phase genes during meiotic differentiation. In this study, we sought to gain insight into the mechanism by which Cuf2 regulates meiotic gene expression. Using a chromatin immunoprecipitation approach, we demonstrate that Cuf2 is specifically associated with promoters of both activated and repressed target genes, in a time-dependent manner. In case of the *fzr1*^*+*^ gene whose transcription is positively affected by Cuf2, promoter occupancy by Cuf2 results in a concomitant increased association of RNA polymerase II along its coding region. In marked contrast, association of RNA polymerase II with chromatin decreases when Cuf2 negatively regulates target gene expression such as *wtf13*^*+*^. Although Cuf2 operates through a transcriptional mechanism, it is unable to perform its function in the absence of the Mei4 transcription factor, which is a member of the conserved forkhead protein family. Using coimmunoprecipitation experiments, results showed that Cuf2 is a binding partner of Mei4. Bimolecular fluorescence complementation experiments brought further evidence that an association between Cuf2 and Mei4 occurs in the nucleus. Analysis of *fzr1*^*+*^ promoter regions revealed that two FLEX-like elements, which are bound by the transcription factor Mei4, are required for chromatin occupancy by Cuf2. Together, results reported here revealed that Cuf2 and Mei4 co-regulate the timely expression of middle-phase genes during meiosis.

## Introduction

Meiosis is the cellular division process by which diploid precursor cells produce haploid gametes [1]. In the fission yeast *Schizosaccharomyces pombe*, diploid cells are formed by conjugation of two haploid cells of opposite mating types (*h*^*+*^*/h*^*-*^) [2]. Following conjugation, the two haploid nuclei fuse and that subsequently induce premeiotic replication of chromosomal DNA. Chromosome duplication results in sets of homologous chromosomes that undergo pairing and recombination. Following this process, homologous chromosomes and sister chromatids are successively segregated during the first (MI) and second (MII) meiotic divisions, respectively. These divisions are followed by forespore membrane biogenesis and generation of four mature haploid spores that are enclosed into an ascus.

Diploid germ cells are present in a very small number in mammalian systems and that makes molecular research studies that rely on substantial sample volumes a formidable task [3]. Moreover, cells obtained from animal models (e.g. vitamin A-deficient mice) and tissue co-culture cells (e.g. Sertoli cells with germ cells) are very difficult to synchronize for their entry into meiosis. This inherent problem represents a major hurdle to investigate the molecular mechanisms that control initiation, progression, and termination of the meiotic program [4, 5]. Over the last few decades, the use of model organisms has proved to be a rewarding avenue to understand meiotic highlights [6]. In this connection, *Schizosaccharomyces pombe* has become a particularly attractive tool, mainly because the development of temperature-sensitive strains has made possible synchronization of cells prior to their entry into the meiotic program [7, 8]. For instance, a fission yeast strain harboring the *pat1-114* mutation produces a temperature-sensitive Pat1 kinase. When *pat1-114* cells undergo a transition from low (25°C) to elevated (34°C) temperature, the Pat1 kinase is readily inactivated and that triggers a cell cycle switch from mitosis to meiosis in a highly efficient and synchronous fashion [9, 10].

Initiation, progression and termination of meiosis are associated with marked changes in gene expression. In the case of fission yeast, transcriptional profiles of the meiotic cell cycle have defined four successive waves of gene expression that are mainly controlled by transcription factor cascades and feedback interactions between these regulators [8, 11]. First, in response to nutritional changes, Ste11 activates expression of pheromone- and starvation-responsive genes, which function in meiosis initiation and commitment. Subsequently, transcription of the nutrient-responsive genes is repressed by Rep1, which in turn activates expression of early-phase genes that are involved in premeiotic S-phase. After homologous recombination completed, Mei4 represses the expression of early-phase genes while it activates transcription of middle-phase genes, which carry out meiotic nuclear divisions and early steps of spore formation [12]. Fine mapping analysis of several promoter regions of genes under the control of Mei4 have identified a consensus cis-acting element, denoted FLEX, which contains a heptamer core (G/A)TAAA(C/T)A to which Mei4 binds [12, 13]. Furthermore, a AACA 3’ flanking sequence is often found immediately downstream of the FLEX core sequence [12]. Finally, late-phase genes are responsible for spore maturation and are essentially activated through a combination of different transcription factors, including Rev2, Atf21 and Atf31 [8, 11].

In addition to these major meiotic activators, other regulators have pivotal roles in regulating meiotic gene expression. During middle-phase meiosis, Mei4 induces the expression of *cuf2*^*+*^, which encodes for a copper fist-like nuclear regulator that is exclusively expressed during meiosis [11, 14]. Within its N-terminal portion, Cuf2 possesses a putative Zn^2+^-coordinating module that in copper-sensing transcription factors makes contact with DNA minor groove [15, 16]. Despite this structural feature, experiments undertaken to establish a role for Cuf2 in the regulation of copper homeostasis were inconclusive [14]. However, it was shown that cells lacking Cuf2 exhibited an elevated and sustained expression of several middle-phase genes (e.g. *wtf13*^+^) that persisted even during late meiosis [14]. Results showed that the absence of Cuf2 triggers errors that jeopardize the quality and quantity of haploid gametes, which leads to a dramatic reduction of spore viability [14]. Thus far, however, the molecular mechanism by which Cuf2 represses expression of middle-phase meiotic genes remains unknown.

As a specialized form of cell division, meiosis undergoes through additional layers of regulation. Ubiquitin-mediated proteolysis is involved in meiotic cell-cycle control as one of the post-translational regulatory mechanisms [17]. One of the major E3 ubiquitin ligase that is responsible for timely degradation of cell cycle-regulatory cyclins during the meiotic program is the anaphase-promoting complex/cyclosome (APC/C) [18]. The APC/C activity is exquisitely regulated during meiosis [17]. One of the factor of APC/C regulation is the Fizzy-related protein Fzr1 (also denoted Mfr1), which recognizes and recruits target substrates via its C-terminal WD40 repeat domain [19–21]. Following Fzr1-mediated substrate recruitment in meiosis II, the APC/C complex becomes fully active and triggers rapid degradation of the Cdc13 cyclin at the end of meiosis II, thus ensuring termination of the meiotic division cycle. Although Mei4 up-regulates *fzr1*^*+*^ expression in middle meiosis, recent studies have shown that Cuf2 further enhances its (*fzr1*^*+*^) transcription to ensure timely proteolysis of Cdc13 [11, 22]. Analogous to *fzr1Δ/Δ* cells, a proportion of ~20% of *cuf2Δ/Δ* mutant cells fail to terminate meiosis II and enter into an aberrant third nuclear division, producing aberrant number of nuclei in several asci [22]. Although it was shown that Cuf2 directly enhances *fzr1*^+^ expression through promoter occupancy, the molecular mechanism by which Cuf2 associates with chromatin has not yet been ascertained.

Because of the critical role of Cuf2 during meiosis, we designed a series of experiments to gain further insight into the mechanism by which Cuf2 affects gene expression. Here, we report that a functional Cuf2-TAP occupies *fzr1*^+^ and *wtf13*^+^ promoters *in vivo*. Furthermore, results of ChIP experiments showed that Cuf2 occupies specific regions in the promoter of both target genes and operates through a transcriptional mechanism to either promote or prevent RNA polymerase II occupancy along *fzr1*^+^ and *wtf13*^+^ transcribed regions. Mei4 was found to be required for Cuf2 function in target gene regulation and its ability to associate with *fzr1*^*+*^ and *wtf13*^*+*^ promoters *in vivo*. Further analysis by coimmunoprecipitation and bimolecular fluorescence complementation assays revealed that Cuf2 is a binding partner of Mei4. We found that two DNA binding sites for Mei4 are required for transcriptional regulation of *fzr1*^*+*^ by Cuf2. Taken together, these results suggested that in the presence of Mei4, Cuf2 acts as a co-regulator for transcriptional control of middle-phase meiotic genes.

## Materials and Methods

### Yeast strains and growth conditions

Genotypes of *S*. *pombe* strains used in this study are described in Table 1. Standard methods were used for growth, sexual conjugation and sporulation of cells [2]. Untransformed strains were cultured on yeast extract medium (YES) that was supplemented with 225 mg/L of adenine, histidine, leucine, uracil and lysine. Cells transformed with gene-swap knock-in cassettes were selected on YES medium supplemented with the geneticin antibiotic (G418, 200 μg/ml) (Sigma-Aldrich). When plasmid integration was required, cells were cultured in Edinburgh minimal medium (EMM) lacking the specific nutrients to select cells expressing integrative vectors. *h*^*+*^/*h*^*+*^ *pat1-114/pat1-114* and *h*^*+*^/*h*^*+*^ *pat1-114/pat1-114 cuf2Δ/cuf2Δ* diploid strains were obtained by incubating mid-logarithmic phase cultures of haploid cells with 20 μg/ml carbendazim (Sigma-Aldrich) as described previously [23]. Preparation and synchronization of *pat1-114*/*pat1-114* diploid cells for their entry into meiosis was performed as described previously [14]. In the cases of Western blot, chromatin immunoprecipitation (ChIP) and co-immunoprecipitation (CoIP) experiments, phenylmethylsulfonyl fluoride (PMSF, 1 mM, Roche) was added directly to the cell cultures 15 min before harvesting to protect proteins from proteolysis.

**Table 1:** *S*. *pombe* strains used in this study.

Strain	Genotype	Source
FY435	*h*^*+*^ *his7-366 leu1-32 ura4-Δ18 ade6-M210*	(14)
RAY1	*h*^*+*^ *his7-366 leu1-32 ura4-Δ18 ade6-M210 cuf2Δ*::*KAN*^*r*^	(14)
RAY29	*h*^*+*^ *his7-366 leu1-32 ura4-Δ18 ade6-M210 cuf2Δ*::*loxP mei4Δ*::*KAN*^*r*^	This study
RAY30	*h*^*+*^ *his7-366 leu1-32 ura4-Δ18 ade6-M210 cuf2Δ*::*loxP mei4Δ*::*KAN*^*r*^	This study
	*nmt1(3X)-mei4*^+^-*GFP*::*ade6*^*+*^, *cuf2*^+^::*leu1*^*+*^	
RAY31	*h*^*+*^ *his7-366 leu1-32 ura4-Δ18 ade6-M210 cuf2Δ*::*loxP mei4Δ*::*KAN*^*r*^	This study
	*nmt1(3X)-mei4*^+^-*GFP*::*ade6*^*+*^, *cuf2*^+^-*TAP*::*leu1*^*+*^	
RAY32	*h*^*+*^ *his7-366 leu1-32 ura4-Δ18 ade6-M210 cuf2Δ*::*loxP mei4Δ*::*KAN*^*r*^	This study
	*nmt1(3X)-mei4*^+^-*GFP*::*ade6*^*+*^, *TAP alone*::*leu1*^*+*^	
RAY33	*h*^*+*^ *his7-366 leu1-32 ura4-Δ18 ade6-M210 cuf2Δ*::*loxP mei4Δ*::*KAN*^*r*^	This study
	*nmt1(3X)-mei4*^+^-*VC*::*ade6*^*+*^, *cuf2*^+^-*VN*::*leu1*^*+*^	
RAY34	*h*^*+*^ *his7-366 leu1-32 ura4-Δ18 ade6-M210 cuf2Δ*::*loxP mei4Δ*::*KAN*^*r*^	This study
	*nmt1(3X)-mei4*^+^-*VC*::*ade6*^*+*^, *VN-fep1*^+^::*leu1*^*+*^	
JB484	*h*^*+*^ *pat1-114 ade6-M210*	(14)
RAY12	*h*^*+*^ *pat1-114 ade6-M210 cuf2Δ*::*KAN*^*r*^	(14)
RAY16	*h*^*+*^ *pat1-114 ade6-M210 cuf2Δ*::*KAN*^*r*^ *cuf2*^*+*^::*ade6*^*+*^	(14)
RAY17	*h*^*+*^ *pat1-114 ade6-M210 cuf2Δ*::*KAN*^*r*^ *cuf2*^*+*^*-TAP*::*ade6*^*+*^	(14)
RAY35	*h*^*+*^ *pat1-114 ade6-M210 cuf2Δ*::*KAN*^*r*^ *nmt1(3X)cuf2*^*+*^::*ade6*^*+*^	This study
RAY36	*h*^*+*^ *pat1-114 ade6-M210 cuf2Δ*::*KAN*^*r*^ *nmt1(3X)cuf2*^*+*^*-TAP*::*ade6*^*+*^	This study
RAY14	*h*^*+*^ *pat1-114 ade6-M210 mei4Δ*::*KAN*^*r*^	(14)
RAY37	*h*^*+*^ *pat1-114 ade6-M210 mei4Δ*::*KAN*^*r*^ *mei4*^*+*^::*ade6*^*+*^	This study
RAY38	*h*^*+*^ *pat1-114 ade6-M210 mei4Δ*::*loxP mei4*^*+*^*-TAP*::*ade6*^*+*^	This study
RAY39	*h*^*+*^ *pat1-114 ade6-M210 cuf2Δ*::*loxP mei4Δ*::*KAN*^*r*^	This study
RAY40	*h*^*+*^ *pat1-114 ade6-M210 cuf2Δ*::*loxP mei4Δ*::*KAN*^*r*^ *cuf2*^*+*^*-TAP*::*ade6*^*+*^	This study
RAY41	*h*^*+*^ *pat1-114 ade6-M210 cuf2Δ*::*loxP mei4Δ*::*KAN*^*r*^ *nmt1(3X)cuf2*^*+*^*-TAP*::*ade6*^*+*^	This study
RAY42	*h*^*+*^ *pat1-114 ade6-M210 cuf2Δ*::*loxP mei4Δ*::*loxP cuf2*^*+*^*-TAP*::*ade6*^*+*^	This study
	*-370fzr1*^*+*^::*KAN*^*r*^	
RAY43	*h*^*+*^ *pat1-114 ade6-M210 cuf2Δ*::*loxP mei4Δ*::*loxP cuf2*^*+*^*-TAP*::*ade6*^*+*^	This study
	*-370FLEX(1–2)ΔΔfzr1*^*+*^::*KAN*^*r*^	
RAY44	*h*^*+*^ *pat1-114 ade6-M210 cuf2Δ*::*loxP mei4Δ*::*loxP cuf2*^*+*^*-TAP*::*ade6*^*+*^	This study
	*-156fzr1*^*+*^::*KAN*^*r*^	
RAY45	*h*^*+*^ *pat1-114 ade6-M210 mei4Δ*::*loxP mei4*^*+*^*-TAP*::*ade6*^*+*^	This study
	*-370 fzr1*^*+*^::*KAN*^*r*^	
RAY46	*h*^*+*^ *pat1-114 ade6-M210 mei4Δ*::*loxP mei4*^*+*^*-TAP*::*ade6*^*+*^	This study
	*-370 FLEX(1–2)ΔΔfzr1*^*+*^::*KAN*^*r*^	
RAY47	*h*^*+*^ *pat1-114 ade6-M210 mei4Δ*::*loxP mei4*^*+*^*-TAP*::*ade6*^*+*^	This study

### Ectopic Mei4-driven meiosis

The thiamine-repressible *nmt1*^+^ promoter (from pREP3X or pREP41X) [24] was cloned immediately upstream of the *mei4*^*+*^ gene. Activation of *nmt1*^+^ fostered production of Mei4 in vegetative cells. Several middle-phase meiotic genes were ectopically expressed in vegetative cells following Mei4 induction, as described previously [11, 25, 26]. Cells expressing an integrated *nmt1*^*+*^ *3X or 41X mei4*^*+*^*-GFP* or *-VC* allele were grown in the presence of 5 μM thiamine. At mid-logarithmic phase, cells were washed twice and incubated for 16, 18 and 20 h in thiamine-free media. At these time points, cells were harvested to carry out various analysis, including RNase protection, ChIP and BiFC assays.

### *cuf2*^*+*^ and *mei4*^*+*^ plasmids

The intron-less DNA segment corresponding to *cuf2*^*+*^ cDNA was generated by a strategy described previously [14]. pBP-500*cuf2*^+^, pBP-500*cuf2*^+^-*TAP*, pJK-500*cuf2*^+^ and pJK-500*cuf2*^+^-TAP plasmids have been described previously [14]. PCR amplification of the *mei4*^*+*^ gene was performed using primers designed to generate PstI and XmaI restriction sites at the upstream and downstream termini of the open reading frame, respectively. The PCR product was digested with PstI and XmaI and swapped for the equivalent DNA restriction fragment in pBP-500*cuf2*^*+*^*-TAP* to generate pBP-500*mei4*^*+*^*-TAP*. Subsequently, the *cuf2*^*+*^ promoter was replaced with the *mei4*^*+*^ promoter (regulatory region up to position -500 from the initiator codon) using the ApaI and PstI sites. The resulting construct allowed expression of a TAP-tagged Mei4 protein under the control of its own promoter in meiotic cells. DNA fragments corresponding to *nmt1*^*+*^ 3X and 41X promoters were isolated from pSP1*nmt1*^*+*^3X-*cuf1*^*+*^*-GFP* and pSP1*nmt1*^*+*^41X-*cuf1*^*+*^*-GFP* [27] using ApaI and PstI restriction enzymes. Purified fragments were inserted into pBP-500*cuf2*^+^, pBP-500*cuf2*^+^-*TAP*, and pBP-500*mei4*^*+*^*-TAP* from which the ApaI-PstI *cuf2*^*+*^ or *mei4*^*+*^ promoter fragment had previously been removed. The resulting plasmids were denoted pBP*nmt1*^*+*^*3X-cuf2*^+^, pBP*nmt1*^*+*^*3X-cuf2*^+^-*TAP*, pBP*nmt1*^*+*^*3X-mei4*^*+*^*-TAP*, pBP*nmt1*^*+*^*41X-cuf2*^+^, pBP*nmt1*^*+*^*41X-cuf2*^+^-*TAP*, and pBP*nmt1*^*+*^*41X-mei4*^*+*^*-TAP*. Plasmids pBP*nmt1*^*+*^*3X-mei4*^*+*^*-TAP* and pBP*nmt1*^*+*^*41X-mei4*^*+*^*-TAP* were digested with XmaI and SacI to remove their TAP coding sequences. A XmaI-SacI PCR-amplified fragment containing the coding region of GFP was then inserted in-frame to the 3’ terminal region of *mei4*^*+*^ to generate plasmids pBP*nmt1*^*+*^*3X-mei4*^*+*^*-GFP* and pBP*nmt1*^*+*^*41X-mei4*^*+*^*-GFP*. Coding regions of Venus N-terminal (VN) and C-terminal (VC) fragments were amplified by PCR from pSP1*ctr5*^*+*^*-VN* and pBP*ctr4*^*+*^*-VC* [28] using primers that added unique XmaI and SacI restriction sites. Subsequently, purified XmaI-SacI DNA fragments (from VN or VC) were exchanged with the XmaI-SacI DNA regions in plasmids pJK-500*cuf2*^+^-TAP and pBP*nmt1*^*+*^*3X (or 41X)-mei4*^*+*^*-TAP* to generate pJK-500*cuf2*^*+*^*-VN* and pBP*nmt1*^*+*^*3X (or 41X)-mei4*^*+*^*-VC*, respectively. Plasmid pJK*VN-fep1*^*+*^, which was used as a control of interaction specificity, has been described previously [29]. The TAP coding sequence was amplified by PCR using primers that added XmaI and SacI unique restriction sites. The resulting DNA fragment was inserted into pJK-500*cuf2*^*+*^-TAP from which the *cuf2*^+^-TAP fragment had been removed using the XmaI-SacI restriction sites to generate the pJK-500*TAP* alone, which was used as a control in CoIP experiments.

### Mutated elements in the promoter region of *fzr1*^*+*^

We engineered strains in which two FLEX elements were mutated in the promoter region of *fzr1*^+^. First, a *fzr1*^+^ promoter region encompassing positions -810 to -661 (with respect to A of the ATG codon of *fzr1*^+^) was amplified by PCR and then inserted into pKSloxP-KAN-loxP at the NotI and EcoRI sites. This plasmid was designated pKS*fzr1*^-810-661^loxP-KAN-loxP. Second, a 1355-bp SalI-Asp718 fragment from the *fzr1*^+^ locus (starting at -370 before the start codon up to +985) was amplified and cloned into the same sites of pKS*fzr1*^-810-661^loxP-KAN-loxP. The resulting plasmid was named pKS*fzr1*^-810-661^loxP-KAN-loxP*fzr1*^-370-+985^. The *fzr1*^+^ promoter region (positions -370 to -1) contained two FLEX sequences (positions -295 to -288 and -208 to -198). These two FLEX elements were either left unchanged (wild-type) or mutated using a PCR overlap extension method [30]. The plasmid containing mutated FLEX sequences was denoted pKS*fzr1*^-810-661^loxP-KAN-loxP*fzr1*^-370-+985^FLEX 1–2 mut. The two plasmids, pKS*fzr1*^-810-661^loxP-KAN-loxP*fzr1*^-370-+985^ and pKS*fzr1*^-810-661^loxP-KAN-loxP*fzr1*^-370-+985^FLEX 1–2 mut, were subsequently digested with NotI and Asp718 (for which the restriction sites are unique and found at positions -810 and +985, respectively) to produce a DNA fragment that allowed homologous integration of wild-type or mutated FLEX elements at the chromosomal locus of *fzr1*^+^, thereby generating a FLEX-containing or FLEX-less *fzr1*^+^ promoter.

### RNA isolation and analysis

Total RNA was extracted using a standard hot phenol method [31]. Gene expression profiles were analyzed using RNase protection assays as described previously [32]. Plasmids pSK*fzr1*^*+*^, pSK*wtf13*^*+*^, pSK*mei4*^*+*^ and pSK*act1*^*+*^ were used to produce antisense RNA probes that served to determine *fzr1*^*+*^, *wtf13*^*+*^, *mei4*^*+*^, and *act1*^*+*^ mRNA levels, respectively. A short coding sequence was amplified by PCR in the case of each gene (Table 2). Primers were designed to generate BamHI and EcoRI sites at the ends of the amplified fragments. Each of these DNA fragments was digested with BamHI and EcoRI and cloned into the same restriction sites of pBluescript SK (Stratagene). ^32^P-labeled anti-sense RNA probes were generated using BamHI-linearized plasmids (pSK*fzr1*^*+*^, pSK*wtf13*^*+*^, pSK*mei4*^*+*^ and pSK*act1*^*+*^), [α-^32^P]UTP and T7 RNA polymerase as described previously [32]. Riboprobe length and hybridization positions relative to the A of the initiator codon of each gene are listed in Table 2. *act1*^*+*^ mRNA was probed as an internal control for normalization during quantification of the RNase protection products.

**Table 2:** Riboprobes used to detect steady-state levels of transcripts.

Gene ID	Gene name length (bp)	Riboprobe initiator codon	Position relative to	Source or reference
*SPBC1198*.*12*	*fzr1*^*+*^*/mfr1*^*+*^	199	+115 to +314	This study
*SPBC32H8*.*11*	*mei4*^*+*^	200	+171 to +371	(14)
*SPCC162*.*04c*	*wtf13*^*+*^	177	+374 to +551	This study
*SPBC32H8*.*12c*	*act1*^*+*^	151	+334 to +485	(14)

### Protein extraction and analysis

To determine the steady-state protein levels of Cuf2-TAP and α-tubulin, whole cell extracts were prepared using a trichloroacetic acid (TCA) extraction method [33]. Equal amounts of each sample preparation were resuspended in sodium dodecyl sulfate loading buffer and proteins were resolved by electrophoresis on 8-% sodium dodecyl sulfate-polyacrylamide gels. Proteins were then electroblotted onto nitrocellulose membranes for 1 h at 4°C. Membranes were blocked by treatment (2 h, at 4°C) with 5% powdered skim milk (Difco) in TBS (10 mM Tris-HCl, pH 7.4, 150 mM NaCl, 1% bovine serum albumin) containing 0.1% Tween 20 (TBST). Following washings with TBST, membranes were incubated with primary antibodies in 1% powdered skim milk in TBST for 16 h at 4°C. The following antibodies were used for immunodetection of Cuf2-TAP and α-tubulin: polyclonal anti-mouse IgG antibody (ICN Biomedicals) and monoclonal anti-α-tubulin antibody (clone B-5-1-2; Sigma-Aldrich), respectively. Following incubation, the membranes were washed and incubated with the appropriate horseradish peroxidase-conjugated secondary antibodies (Amersham Biosciences), developed with enhanced chemiluminescence (ECL) reagents (Amersham Biosciences), and visualized by chemiluminescence using an ImageQuant LAS 4000 instrument (GE Healthcare) equipped with a Fujifilm High Sensitivity F0.85 43 mm camera.

### ChIP experiments

In vivo chemical cross-linking of proteins was performed by incubating cell cultures in the presence of 1% formaldehyde for 20 min. After formaldehyde-mediated cross-links and neutralization with glycine, cell lysates were prepared by glass bead disruption in lysis buffer containing 100 mM HEPES-KOH pH 7.5, 1% Triton X-100, 0.1% Na-deoxycholate, 1 mM EDTA, 140 mM NaCl, 2X cOmplete ULTRA Tablets (protease inhibitors, Roche), 1 mM phenylmethylsulfonyl fluoride, 50 mM NaF and 0.2 mM Na_3_VO_4_, as described previously [34]. Samples were subsequently sonicated 10 times (10 s cycles at 20 amplitude microns [20%]) using a Branson 450 sonicator in order to shear chromatin DNA into fragments of ~500 to 1000 bp. Immunoprecipitation of Cuf2-TAP and Mei4-TAP proteins bound to chromatin was performed using immunoglobin G (IgG)-Sepharose beads. In the case of the RNA polymerase II, immunoprecipitation was carried out using protein G sepharose beads that were coupled to a monoclonal anti-Rpb1 antibody (clone 8WG16, Covance), which specifically recognizes a repeated seven-residue motif (YSPTSTS) located at the C-terminus of Rpb1, termed the C-terminal domain (CTD). Manipulation of beads, including washings and elution, reversed cross-linking, and DNA precipitation were performed as described previously [35, 36]. Quantification of immunoprecipitated DNA was carried out by real-time PCR (qPCR) using different sets of primers that spanned *fzr1*^*+*^, *wtf13*^*+*^, and *mei4*^*+*^ promoter regions. Cuf2-TAP, Mei4-TAP or RNA polymerase II density at *fzr1*^*+*^, *wtf13*^*+*^, and *mei4*^*+*^ promoters was calculated as the enrichment of specific genomic *fzr1*^*+*^, *wtf13*^*+*^, and *mei4*^*+*^ promoter regions relative to a 18S ribosomal DNA coding region. Primers that were used for qPCR analysis are listed in Table 3. Each qPCR was run in triplicate using Perfecta SYBR Green Fast mix (Quanta) on a LightCycler 96 Real-Time PCR instrument (Roche). All ChIP experiments were repeated at least three times using independent chromatin preparations.

**Table 3:** Primers used for qPCR analyses in ChIP experiments.

Gene name	Primer set	Position relative to initiator codon	Amplicon length (bp)	Source
*fzr1*^*+*^*/mfr1*^*+*^	A	-1032/-896	137	This study
	B	-649/-505	145	This study
	C	-398/-290	109	This study
	D	-130/-31	100	This study
	ORF A	278/416	139	This study
	ORF B	765/890	110	This study
	ORF C	1038/1187	126	This study
	3’-UTR A	+112/+210	99	This study
*wtf13*^+^	A	-1047/-903	145	This study
	B	-652/-575	78	This study
	C	-400/-250	151	This study
	D	-184/-88	97	This study
	ORF A	278/416	139	This study
	ORF B	1440/1547	108	This study
	3’-UTR A	+528/+632	105	This study
*mei4*^+^	A	-899/-747	153	This study
	B	-570/-445	126	This study
	C	-222/-124	99	This study
	ORF A	16/148	133	This study
	ORF B	1082/1203	122	This study

### Co-immunoprecipitation experiments

To determine whether Cuf2 interacted with Mei4 in *S*. *pombe*, *cuf2Δ mei4Δ* cells were co-transformed with pBP*nmt1*^*+*^*-mei4*^*+*^*-GFP* and pJK-500*cuf2*^*+*^*-TAP*; pBP*nmt1*^*+*^*-mei4*^*+*^*-GFP* and pJK-500*cuf2*^*+*^ or, pBP*nmt1*^*+*^*-mei4*^*+*^*-GFP* and pJK-500*TAP* alone. Mid-logarithmic cell cultures were grown in thiamine-free medium to induce mitotic expression of Mei4, therefore fostering artificial expression of middle-phase meiotic genes in vegetative cells. Total cell lysates were obtained by glass bead disruption in lysis buffer (10 mM Tris-HCl (pH 7.9), 0.1% Triton X-100, 0.5 mM EDTA, 20% glycerol, 100 mM NaCl, 1 mM phenylmethylsulfonyl fluoride, 50 mM NaF and 0.2 mM Na_3_VO_4_) containing a mixture of 2X protease inhibitors (cOmplete ULTRA Tablets, Roche). The mixture was centrifuged (13,000 rpm) and equal amounts of proteins (10 mg) were added to 25-μl bed volumes of IgG Sepharose 6 Fast-Flow beads (GE Healthcare) and the suspensions were mixed by inversion for 2 h at 4°C. The beads were washed three times with 1 ml of lysis buffer and then transferred to fresh microtubes prior to a final wash. The immunoprecipitates were resuspended in 120 μl of sodium dodecyl sulfate loading buffer and proteins resolved by electrophoresis on 8% sodium dodecyl sulfate-polyacrylamide gels. The following antibodies were used: polyclonal anti-mouse IgG antibody (ICN Biomedicals), polyclonal anti-GFP antibody (clone FL; Santa Cruz Biotechnology) and monoclonal anti-α-tubulin antibody (clone B-5-1-2; Sigma-Aldrich) in Western blot analysis of Cuf2-TAP, TAP alone, Mei4-GFP, and α-tubulin. Membranes were developed using Amersham ECL Prime Western blotting detection reagent (GE Healthcare).

### Bimolecular fluorescence complementation (BiFC) analysis

*cuf2Δ mei4Δ* or *fep1Δ mei4Δ* double mutant cells co-expressing Mei4-VC and Cuf2-VN or Mei4-VC and VN-Fep1 fusion proteins were grown to mid-log phase in EMM containing 5 μM thiamine. Cells were then washed twice, diluted to an *A*_*600*_ of ~0.03 in EMM without thiamine, and then cultured for 18 h. After Mei4 induction and artificial expression of middle-phase meiotic genes in vegetative cells, fluorescence and differential interference contrast images were viewed using an Eclipse E800 epifluorescent microscope (Nikon, Melville, NY) equipped with an ORCA ER digital cooled camera (Hamamatsu, Bridgewater, NJ). BiFC signals were visualized using a magnification of X1,000 with a transmission window of 465 to 495 nm, whereas chromosomal material (stained with Hoechst 33342 dye) was detected with a window of 340 to 380 nm. Cell fields shown in this study represent a minimum of five independent experiments. The merged images were obtained using the Simple PCI software, version 5.3.0.1102 (Compix, Sewickly, PA).

## Results

### Cuf2 is required for the timely expression of *fzr1*^*+*^ and *wtf13*^*+*^ during meiosis

**Fig 1:**
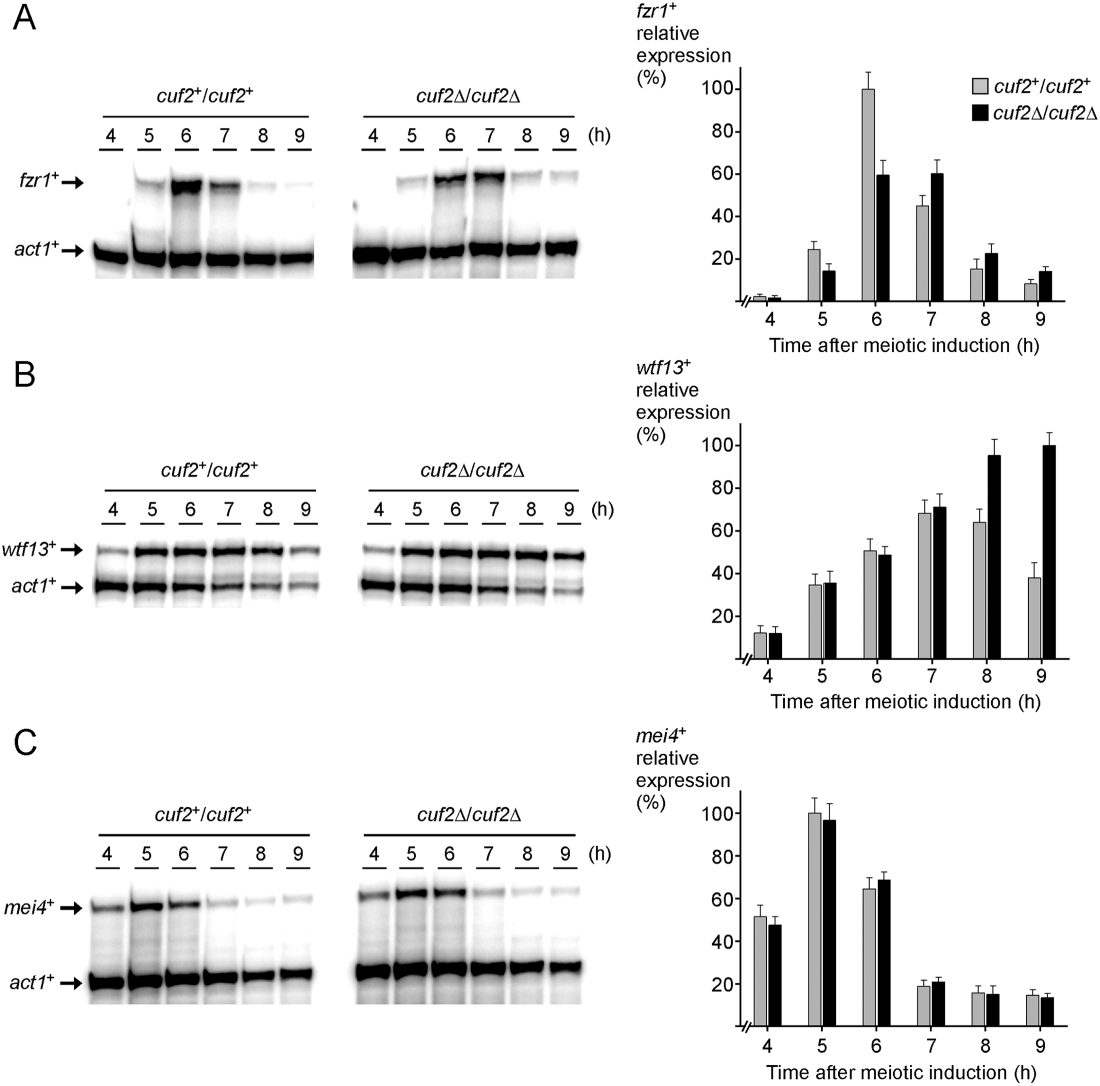
Cuf2 coordinates timely expression of *fzr1*^*+*^ and *wtf13*^*+*^ during meiosis. Cultures of *pat1-114/pat1-114 cuf2*^*+*^*/cuf2*^*+*^ and *pat1-114/pat1-114 cuf2Δ/cuf2Δ* cells were induced to initiate and proceed through meiosis. At the indicated times after meiotic induction, *fzr1*^*+*^ (panel A), *wtf13*^*+*^ (panel B), and *mei4*^*+*^ (panel C) mRNA levels were analyzed by RNase protection assays. For each panel, graphic representation of quantification of the results of three independent RNase protection assays is depicted, including the experiment shown on left side. Values indicate the normalized *fzr1*^*+*^, *wtf13*^*+*^, and *mei4*^*+*^ transcript levels relative to *act1*^*+*^ mRNA levels. Histogram values are shown as averages ± S.D.

In a previous study, we used a genome-wide DNA microarray approach and found that 9 h after meiotic induction, a total of 545 genes were differentially expressed in cells lacking Cuf2 (*cuf2*Δ/*cuf2*Δ) compared to control cells expressing Cuf2 (*cuf2*^+^/*cuf2*^+^) [14]. Among these genes, 247 were expressed at higher levels in *cuf2*Δ/*cuf2*Δ mutant cells, including 144 genes that were annotated as meiosis-specific middle genes. Comprehensive expression profile analyses of 4 middle-phase meiotic genes, including *wtf13*^+^, established that Cuf2 was required for gene repression at the onset of late meiosis [14]. More recently, Yamamoto’s group has uncovered from a genetic screen that the meiotic fizzy-related gene 1, denoted *fzr1*^+^/*mfr1*^*+*^, was a critical target gene of Cuf2 [22]. During the transition phase from meiotic division I to II, Cuf2 enhances *fzr1*^*+*^ gene expression, thereby producing sufficient quantities of Fzr1 protein that is necessary to terminate the meiotic division cycle [22]. Since our previous DNA microarray experiments were performed only after completion of the meiotic divisions, our results did not highlight the effect of *cuf2Δ/Δ* deletion on the expression of *fzr1*^*+*^. To validate that indeed, Cuf2 activates *fzr1*^*+*^ transcription while it also acts as a negative regulator for the expression of other middle-phase genes, RNase protection assays were performed to measure steady-state transcript levels of *fzr1*^*+*^ and *wtf13*^*+*^ in wild-type versus *cuf2Δ/cuf2Δ* cells. Synchronization of wild-type and *cuf2Δ/cuf2Δ* cells was verified by probing mRNA levels of *mei4*^+^. Furthermore, expression of *mei4*^+^ was tested as a control of a middle meiotic gene that is not regulated by Cuf2 [14]. Diploid *pat1-114/pat1-114 cuf2*^*+*^*/cuf2*^*+*^ and *pat1-114/pat1-114 cuf2Δ/cuf2Δ* cells were induced to undergo synchronous meiosis and total RNA was subsequently isolated from culture aliquots taken at different time points. In the case of *cuf2*^*+*^/*cuf2*^*+*^ cells, results showed that *fzr1*^*+*^ mRNAs were absent in early meiosis but reached maximum levels after 6 h of meiotic induction (Fig 1 and unpublished data). This peak of gene expression was followed by a ~2.5-fold reduction of *fzr1*^*+*^ mRNA levels that occurred 7 h after meiotic induction. *fzr1*^*+*^ transcript levels were subsequently repressed within 8–9 h (Fig 1). In the case of *cuf2Δ*/*cuf2Δ* cells, *fzr1*^*+*^ transcript levels were detected after 6 h of meiotic induction but to a lesser extent (~40% less) in comparison with transcript levels observed in *cuf2*^*+*^/*cuf2*^*+*^ cells (Fig 1). In mutant cells, *fzr1*^*+*^ mRNA levels remained elevated and nearly unchanged after 7 h of the meiotic program. Weak *fzr1*^*+*^ mRNA levels were only observed after 8 h of meiotic induction (Fig 1). Expression of *wtf13*^*+*^ was also analyzed as a function of time during meiosis (Fig 1). At the 5-h time point, RNase protection experiments revealed that the expression levels of *wtf13*^*+*^ were increased in a similar manner in both *cuf2*^*+*^/*cuf2*^*+*^ and *cuf2Δ*/*cuf2Δ* cells. However, *wtf13*^*+*^ mRNA levels were ~1.5- and ~2.6-fold higher in *cuf2Δ*/*cuf2Δ* cells as compared to those in control cells after 8 and 9 h of meiotic induction, respectively (Fig 1). These results confirmed that expression of *wtf13*^*+*^ was up-regulated and remained at high steady-state levels even during late meiosis (in *cuf2Δ*/*cuf2Δ*) when its expression normally was reduced (in *cuf2*^*+*^/*cuf2*^*+*^). In the case of mRNA steady-state levels of *mei4*^*+*^, the *cuf2Δ*/*cuf2Δ* mutant did not show any significant effect on the expression of *mei4*^*+*^ as a function of time in meiosis (Fig 1). Taken together, these results showed that Cuf2 functions in the timely activation and repression of *fzr1*^*+*^ and *wtf13*^*+*^, respectively, therefore ensuring the adequate abundance of transcripts at the correct times during meiosis.

### Cuf2 associates with *fzr1*^*+*^ and *wtf13*^*+*^ promoters in vivo

**Fig 2:**
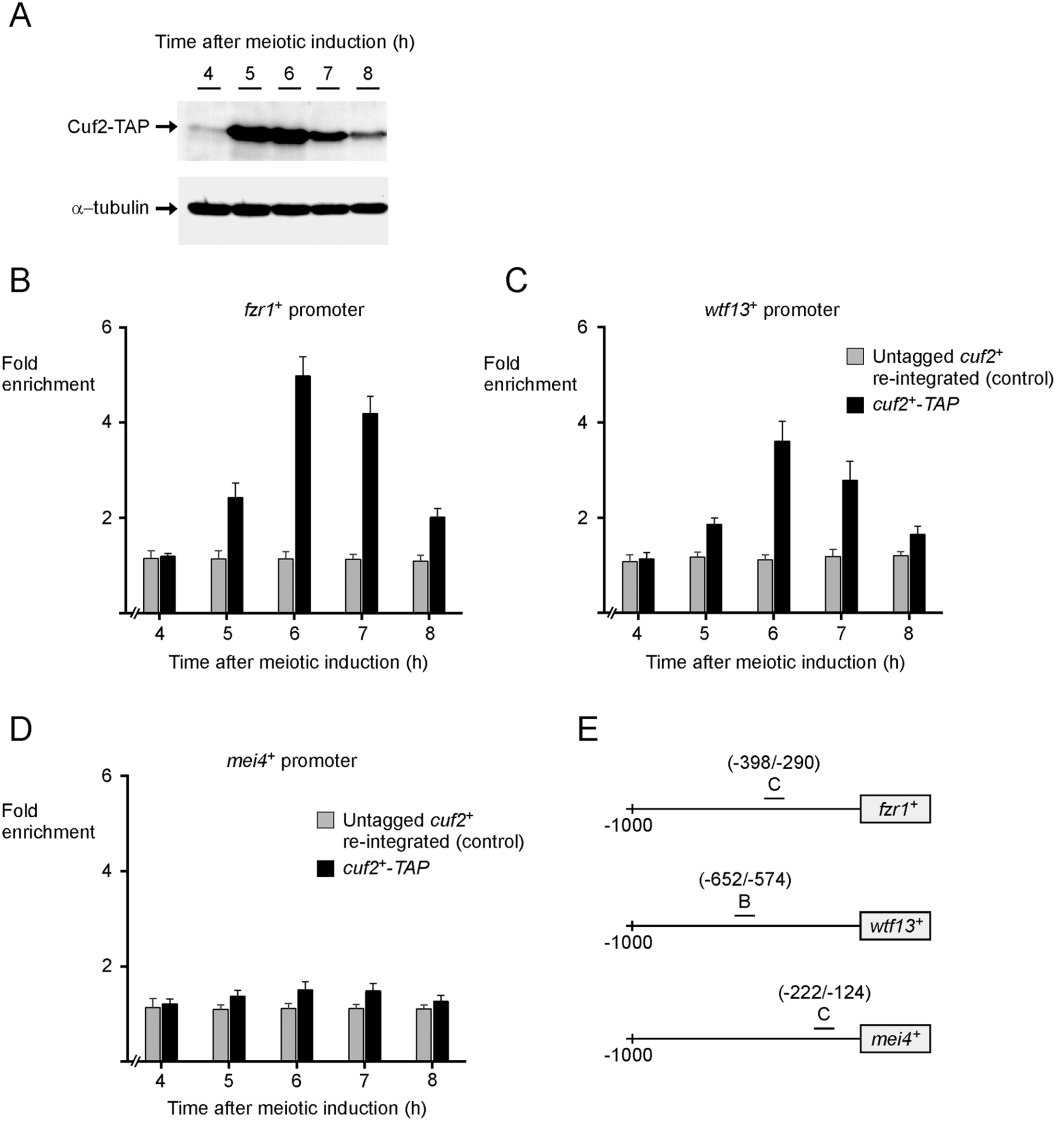
Cuf2 interacts with *fzr1*^*+*^ and *wtf13*^*+*^ promoters mostly in middle-phase meiosis. A, *pat1-114*/*pat1-114 cuf2Δ*/*cuf2Δ* cells expressing Cuf2-TAP were synchronously induced to undergo meiosis. Protein extracts were analyzed by Western blots for steady-state protein levels of Cuf2-TAP and α-tubulin at different time points after meiotic induction. B-D, Synchronized cells described in *panel A* were used for ChIP assays. *pat1-114*/*pat1-114 cuf2Δ*/*cuf2Δ* cells expressing an integrated untagged *cuf2*^*+*^ allele were also synchronized. Following induction of meiosis, chromatin was immunoprecipitated using resin-bound anti-mouse IgG antibodies at the indicated time points. Specific regions of *fzr1*^*+*^, *wtf13*^*+*^, and *mei4*^*+*^ promoters were analyzed by qPCR to determine Cuf2-TAP occupancy. Binding of Cuf2-TAP to promoters was calculated as the enrichment of specific *fzr1*^*+*^, *wtf13*^*+*^, and *mei4*^*+*^ promoter regions relative to a 18S ribosomal DNA coding region. ChIP data were calculated as values of the largest amount of chromatin measured (fold enrichment). Results are shown as the averages ± S.D. of a minimum of three independent experiments. E, Schematic representation of *fzr1*^*+*^, *wtf13*^*+*^, and *mei4*^*+*^ promoter regions. Nucleotide numbers refer to the position relative to the A of the initiator codon of each gene (*fzr1*^*+*^, *wtf13*^*+*^, or *mei4*^*+*^). Underlined capital letters indicate promoter regions that were used for qPCR analysis.

Although it has been shown that Cuf2 activates transcription of *fzr1*^+^ through its association with the *fzr1*^+^ promoter in vivo [22], promoter occupancy by Cuf2 as a function of time remains uncharacterized. Moreover, it remains unclear whether Cuf2 directly represses middle-phase meiotic genes (e.g. *wtf13*^+^). To gain insight into these aspects, *pat1-114*/*pat1-114 cuf2Δ*/*cuf2Δ* cells expressing either an untagged or a functional TAP-tagged version of Cuf2 under the control of its own promoter were synchronized to initiate and to proceed through meiosis. Each hour over a time period of 4 to 8 h after meiotic induction, cells were either fixed by formaldehyde treatment for ChIP assays or harvested and lysed to analyze steady-state levels of Cuf2-TAP. Results showed that Cuf2-TAP was first detected after 5 h of meiotic induction (Fig 2). At this time point (5h) and after 6 and 7 h of meiotic induction, Cuf2-TAP protein levels were elevated. In contrast, at the 8-h time point, results showed that levels of Cuf2-TAP were significantly reduced compared with levels observed after 5 h of meiotic induction. Results from ChIP analysis showed that Cuf2-TAP occupied *fzr1*^+^ and *wtf13*^+^ promoters at maximum levels 6 h after meiotic induction (Fig 2). At this 6-h time point, Cuf2-TAP exhibited 5.0- and 3.8-fold increases in its binding to *fzr1*^+^ and *wtf13*^+^ promoters, respectively (Fig 2). Values obtained were expressed as fold enrichment relative to a control 18S ribosomal DNA coding region that did not exhibit any transcriptional variation as a function of Cuf2 availability. In the case of the association of Cuf2-TAP with the *fzr1*^*+*^ promoter, its level of occupancy was 52%, 16% and 58% lower after 5, 7 and 8 h of meiotic induction, respectively, compared with level observed after 6 h of meiotic induction (Fig 2). Similarly, Cuf2-TAP exhibited a reduced ability to bind *wtf13*^*+*^ promoter after 5 h (52% lower), 7 h (24% lower) and 8 h (58% lower) of meiotic induction as compared with the 6-h time point (Fig 2). Results from ChIP analysis showed that Cuf2-TAP did not associate with the *mei4*^*+*^ promoter. Only very weak levels of *mei4*^*+*^ promoter fragments were immunoprecipitated (Fig 2). These low levels of immunoprecipitated chromatin were similar to the background signals observed when ChIP assays were performed in *cuf2Δ*/*cuf2Δ* cells expressing untagged *cuf2*^*+*^, which had been re-integrated (Fig 2). Taken together, these results suggested that *fzr1*^*+*^ and *wtf13*^*+*^ promoters are occupied by Cuf2 in a time-dependent manner, mostly during the middle-phase of meiosis.

**Fig 3:**
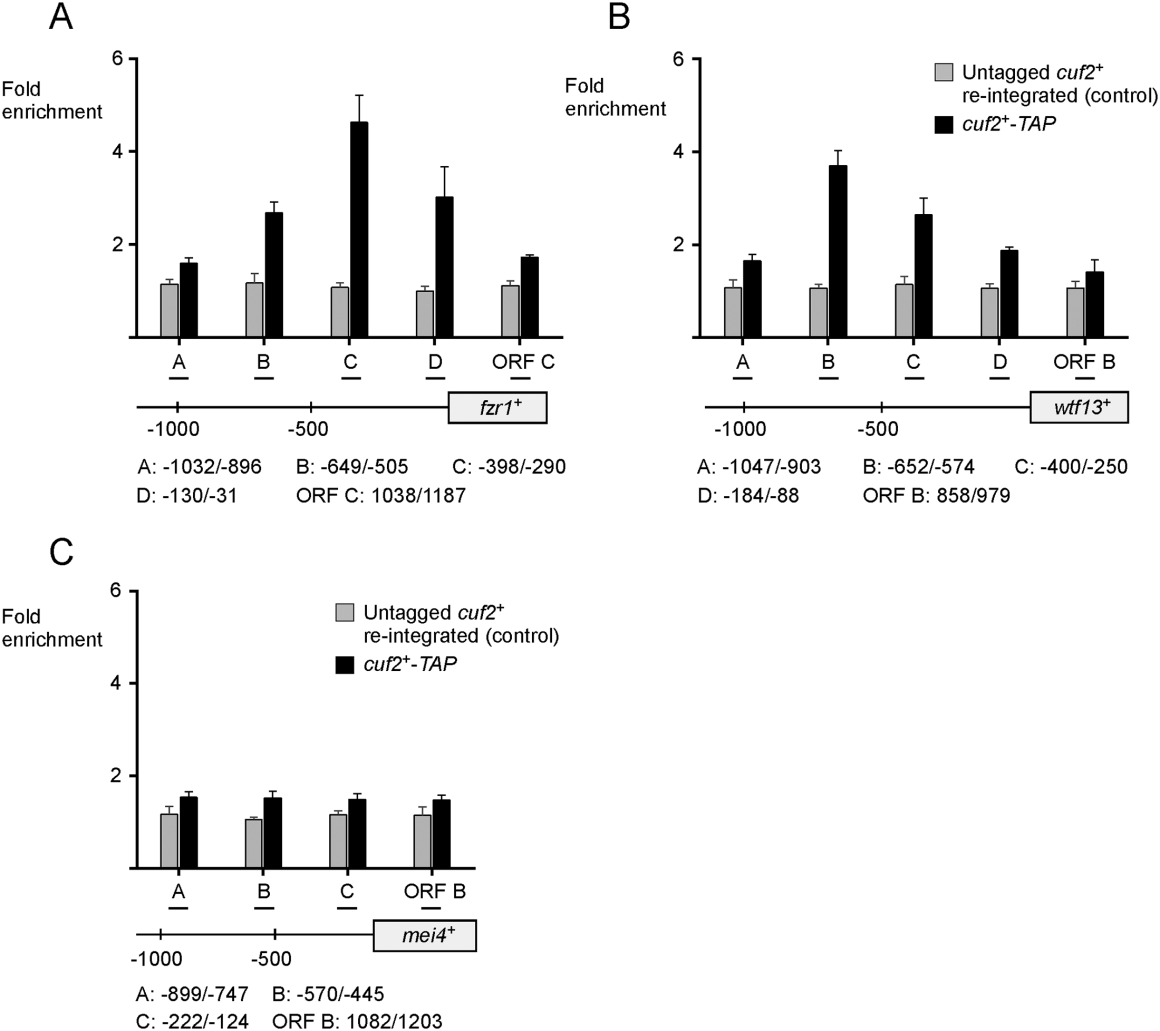
Distribution of Cuf2-occupied regions in chromatin at *fzr1*^*+*^ and *wtf13*^*+*^ loci. *pat1-114*/*pat1-114 cuf2Δ*/*cuf2Δ* cells expressing an integrated untagged or a TAP-tagged *cuf2*^*+*^ allele were synchronously induced to undergo meiosis. After 6 h of meiotic induction, chromatin was immunoprecipitated using anti-mouse IgG antibodies. Following chromatin preparation, specific regions (indicated with underline letters A–D and ORF B/C) of *fzr1*^*+*^ (panel A), *wtf13*^*+*^ (panel B) and *mei4*^*+*^ (panel C) promoters were analyzed by qPCR to determine Cuf2 occupancy. ChIP results are presented as enrichments of specific *fzr1*^*+*^, *wtf13*^*+*^, or *mei4*^*+*^ promoter regions relative to a 18S ribosomal DNA coding region. Data were calculated as values of the largest amount of chromatin measured (fold enrichment) and results are shown as the averages ± S.D. of a minimum of three independent experiments. Nucleotide numbers refer to the position relative to the A of the initiator codon of each gene (*fzr1*^*+*^, *wtf13*^*+*^, or *mei4*^*+*^).

The association of Cuf2-TAP with the *fzr1*^+^ promoter was detected using primers amplifying a DNA region located between positions -398 and -290 (denoted region C) relative to the initiator codon of *fzr1*^+^ (Fig 2). To verify if this region was the best choice to detect binding of Cuf2-TAP to chromatin, other pairs of primers were used that were located farther upstream (positions -1032 to -896 [region A] and -649 to -505 [region B]) or downstream (positions -130 to -31 [region D] and +1038 to +1187 [region ORF C]) from the initiator codon of *fzr1*^+^ (Fig 3). Results of ChIP assays showed that region C produced the highest enrichment of immunoprecipitated chromatin (4.6-fold) in the presence of Cuf2-TAP relative to a 18S ribosomal DNA coding sequence that was used as a negative control (Fig 3). Use of regions B and D gave intermediate enrichment values (2.7- and 3.0-fold, respectively) of immunoprecipitated chromatin by Cuf2-TAP. In contrast, only very low levels of *fzr1*^+^ DNA were detected in immunoprecipitates using regions A (1.6-fold) and ORF C (1.7-fold) (Fig 3). In the case of the interaction of Cuf2-TAP with *wtf13*^+^ promoter, amplification of a DNA region located between positions -652 and -575 (denoted region B) produced the highest enrichment of immunoprecipitated chromatin (3.7-fold) relative to a control 18S ribosomal DNA coding sequence (Fig 3). Other *wtf13*^+^ regions located between positions -1047 to -903 (region A), -400 to -250 (region C), -184 to -88 (region D), and +858 to +979 (region ORF B) (relative to the initiator codon of *wtf13*^+^) were tested. These regions produced weaker enrichments of immunoprecipitated chromatin by Cuf2-TAP (A = 1.7-fold; C = 2.7-fold; D = 1.9-fold; and, ORF B = 1.4-fold) (Fig 3). When we used *mei4*^+^ promoter that is independent of Cuf2 for its regulation, results showed very weak or marginal enrichment of immunoprecipitated chromatin corresponding to different promoter regions (denoted A, B, C, and ORF B) relative to a 18S ribosomal DNA coding sequence that was used as a negative control (Fig 3). Furthermore, results showed that untagged Cuf2 (expressed in *cuf2Δ/cuf2Δ* cells) immunoprecipitated only background levels of *mei4*^*+*^ promoter regions (Fig 3). Taken together, these results identified two regions within *fzr1*^+^ and *wtf13*^+^ promoters that are required for maximal Cuf2-TAP chromatin occupancy.

### Cuf2 modulates RNA polymerase II chromatin occupancy at *fzr1*^*+*^ and *wtf13*^*+*^ transcribed regions

**Fig 4:**
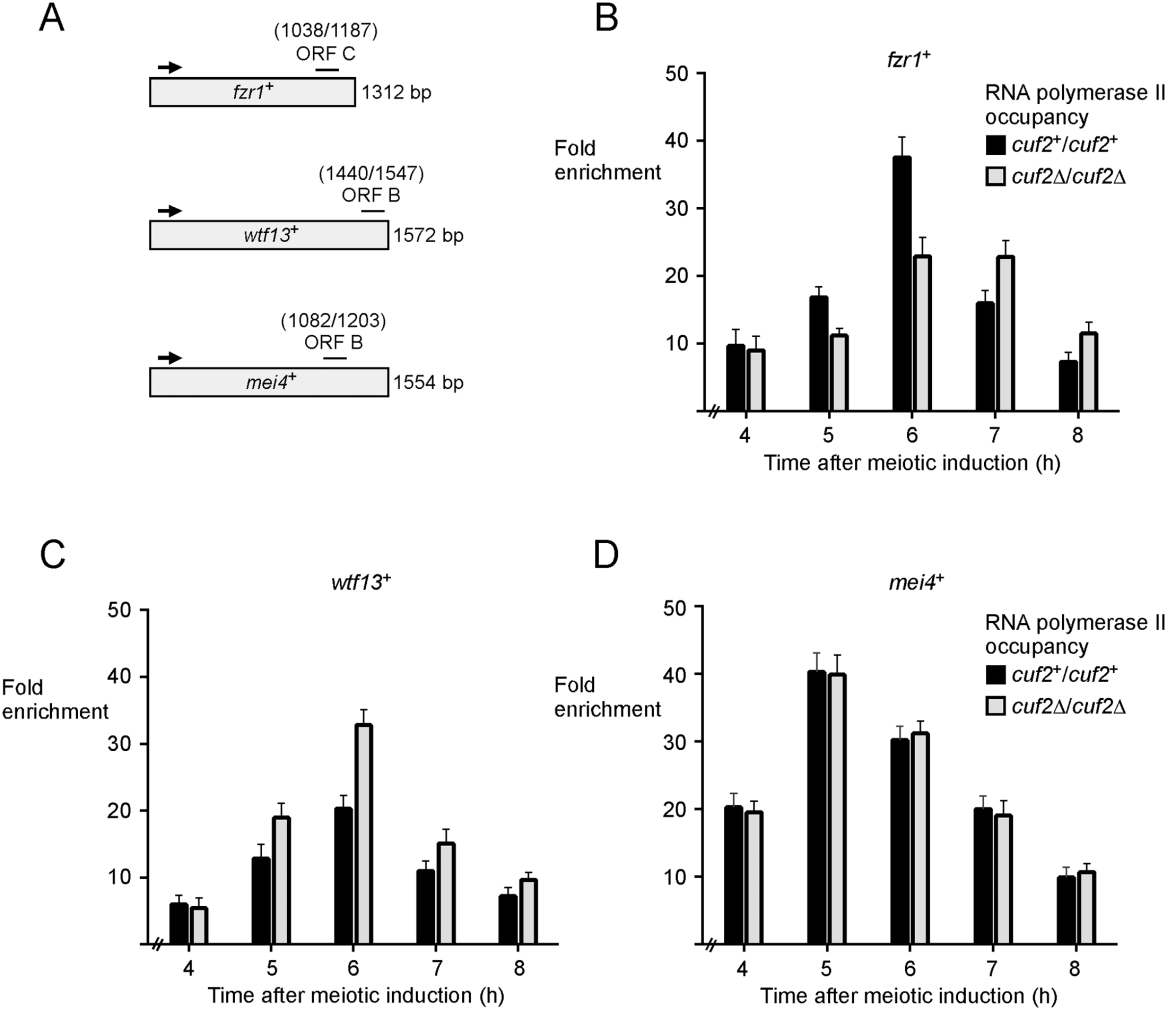
Effect of the presence or absence of Cuf2 on RNA polymerase II chromatin occupancy at *fzr1*^*+*^ and *wtf13*^*+*^ loci. A, Diagram representation of *fzr1*^*+*^, *wtf13*^*+*^, and *mei4*^*+*^ genes in which ORF B and ORF C indicate locations of the primers that were used for qPCR analysis. Arrows indicate the direction of transcription. Nucleotide numbers refer to the position relative to the A of the initiator codon of each gene (*fzr1*^*+*^, *wtf13*^*+*^, or *mei4*^*+*^). B–D, Cultures of *pat1-114/pat1-114 cuf2*^*+*^*/cuf2*^*+*^ and *pat1-114/pat1-114 cuf2Δ/cuf2Δ* cells were induced to initiate and proceed through meiosis. At the indicated times after meiotic induction, chromatin was immunoprecipitated using antibodies against RNA polymerase II CTD. Following chromatin preparation, specific regions of *fzr1*^*+*^, *wtf13*^*+*^, and *mei4*^*+*^ genes were analyzed by qPCR to determine RNA polymerase II occupancy. ChIP results are presented as enrichments of specific coding regions (*fzr1*^*+*^, *wtf13*^*+*^, or *mei4*^*+*^) relative to a 18S ribosomal DNA coding region. Data were calculated as values of the largest amount of chromatin measured (fold enrichment). Results are shown as the averages ± S.D. of a minimum of three independent experiments.

RNA polymerase II (Pol II) is a multi-subunit enzyme that transcribes specific genes into mRNA in eukaryotes [37]. Transcriptional regulators control either positively or negatively the rate of transcription through a myriad of different mechanisms that affect Pol II occupancy at specific coding regions of target genes [38]. We used a Pol II ChIP assay to determine whether Cuf2 regulated meiotic gene expression through a transcriptional mechanism. Analysis of Pol II chromatin occupancy was performed using an anti-Rpb1 antibody that recognizes the heptapeptide sequence YSPTSPS, which is found in multiple copies at the C-terminal domain (CTD) of Pol II [39]. *pat1-114/pat1-114 cuf2*^*+*^*/cuf2*^*+*^ and *pat1-114/pat1-114 cuf2Δ/cuf2Δ* cells were synchronously induced into meiosis and then fixed (formaldehyde) every hour from 4 to 8 h after meiotic induction. Primers that hybridized at the 3’ end of *fzr1*^*+*^ (positions +1038 to +1187), *wtf13*^*+*^ (positions +1440 to +1547), and *mei4*^*+*^ (positions +1082 to +1203) coding regions were used for qPCR analysis (Fig 4). Results showed that in the case of *fzr1*^+^ and *wtf13*^+^, the most significant differences with respect to Pol II chromatin occupancy between *cuf2*^+^/*cuf2*^+^ and *cuf2*Δ/*cuf2*Δ cells were observed 6 h after meiotic induction (Fig 4). In cells expressing Cuf2, association of Pol II with the *fzr1*^*+*^ locus was enriched (37.6-fold) relative to a 18S ribosomal DNA coding region. Under the same growth conditions, *cuf2*Δ/*cuf2*Δ cells exhibited a 22.9-fold enrichment of Pol II, consistent with the interpretation that Cuf2 enhanced Pol II occupancy at the *fzr1*^*+*^ locus (39.1% higher) (Fig 4). In the case of *wtf13*^*+*^, opposite results were observed after 6 h. Levels of Pol II enrichment were higher in cells lacking Cuf2 (32.8-fold) in comparison with those of a *cuf2*^+^/*cuf2*^+^ strain (20.3-fold), revealing that Cuf2 decreased Pol II occupancy at the *wtf13*^*+*^ locus (38.1% lower) (Fig 4). At the 6-h time point, Pol II chromatin occupancy at *mei4*^*+*^ locus remained constant with similar levels of enrichment (30.2- and 31.2-fold) in *cuf2*^+^/*cuf2*^+^ and *cuf2*Δ/*cuf2*Δ cells (Fig 4). In cells expressing Cuf2, results showed that the association of Pol II with the *fzr1*^*+*^ locus was slightly enriched after 5 h of the meiotic program relative to cells lacking Cuf2 (*cuf2*Δ/*cuf2*Δ) (Fig 4). In contrast, after 7 and 8 h of meiotic induction, the association of Pol II with *fzr1*^*+*^ was slightly lower in *cuf2*^+^/*cuf2*^+^ cells than that observed in *cuf2*Δ/*cuf2*Δ cells (Fig 4). In the case of *wtf13*^*+*^, Pol II chromatin occupancy was slightly enriched in cells lacking Cuf2, except at the 4-h time point (Fig 4). Taken together, these results showed that at the 6-h time point of meiosis, Cuf2 promotes Pol II chromatin occupancy at *fzr1*^*+*^, whereas it decreases Pol II chromatin occupancy at *wtf13*^*+*^.

**Fig 5:**
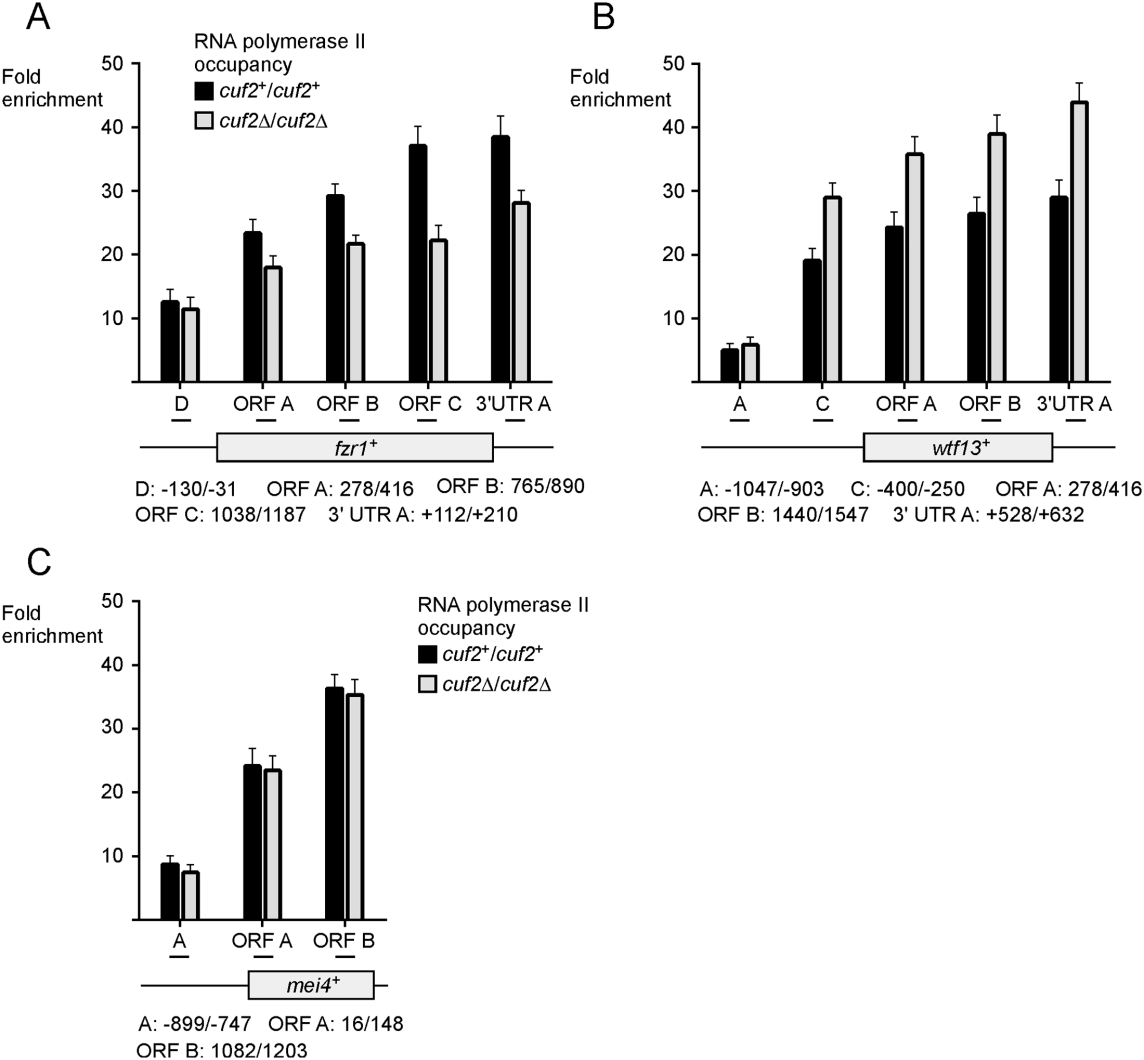
Cuf2 affects RNA polymerase II chromatin occupancy at *fzr1*^*+*^ and *wtf13*^*+*^ genes. *pat1-114/pat1-114 cuf2*^*+*^*/cuf2*^*+*^ and *pat1-114/pat1-114 cuf2Δ/cuf2Δ* strains were synchronously induced into meiosis. At the 6-h meiotic time point, chromatin was immunoprecipitated using RNA polymerase II CTD-specific antibodies. Quantification of immunoprecipitated chromatin was performed by qPCR using primers that allowed amplification of several specific DNA regions (A, C, D, ORF A, ORF B, ORF C, and 3’UTR A), which spanned *fzr1*^*+*^ (panel A), *wtf13*^*+*^ (panel B), and *mei4*^*+*^ (panel C) genes. ChIP results are presented as enrichments of specific noncoding and coding regions (*fzr1*^*+*^, *wtf13*^*+*^, or *mei4*^*+*^) relative to a 18S ribosomal DNA coding region. Data were calculated as values of the largest amount of chromatin measured (fold enrichment). Results are shown as the averages ± S.D. of a minimum of three independent experiments. Arrows indicate the direction of transcription. Nucleotide numbers refer to the position relative to the A of the initiator codon of each gene (*fzr1*^*+*^, *wtf13*^*+*^, or *mei4*^*+*^).

To confirm that indeed Cuf2 affected transcription *per se*, we dissected Pol II chromatin occupancy using primers covering the transcribed regions of *fzr1*^+^, *wtf13*^+^ and *mei4*^+^ in *cuf2*Δ/*cuf2*Δ and *cuf2*^+^/*cuf2*^+^ cells after 6 h of meiotic induction. In the case of the *fzr1*^+^ transcribed region, results showed that Pol II enrichment levels were consistently lower in *cuf2*Δ/*cuf2*Δ cells compared to those observed in *cuf2*^+^/*cuf2*^+^ cells (Fig 5). In contrast, Pol II enrichment levels were higher in *cuf2*Δ/*cuf2*Δ cells compared to those in *cuf2*^+^/*cuf2*^+^ cells within the *wtf13*^+^ transcribed region (Fig 5). As a control, results showed no significant variation in Pol II chromatin occupancy with respect to *mei4*^*+*^ transcribed region regardless of strain genotype. Taken together, results led support to the conclusion that Cuf2 positively or negatively influences meiotic gene expression by way of a transcriptional mechanism which affects Pol II chromatin occupancy at target gene coding regions.

### Cuf2-dependent regulation of *fzr1*^*+*^ and *wtf13*^*+*^ requires the presence of Mei4

**Fig 6:**
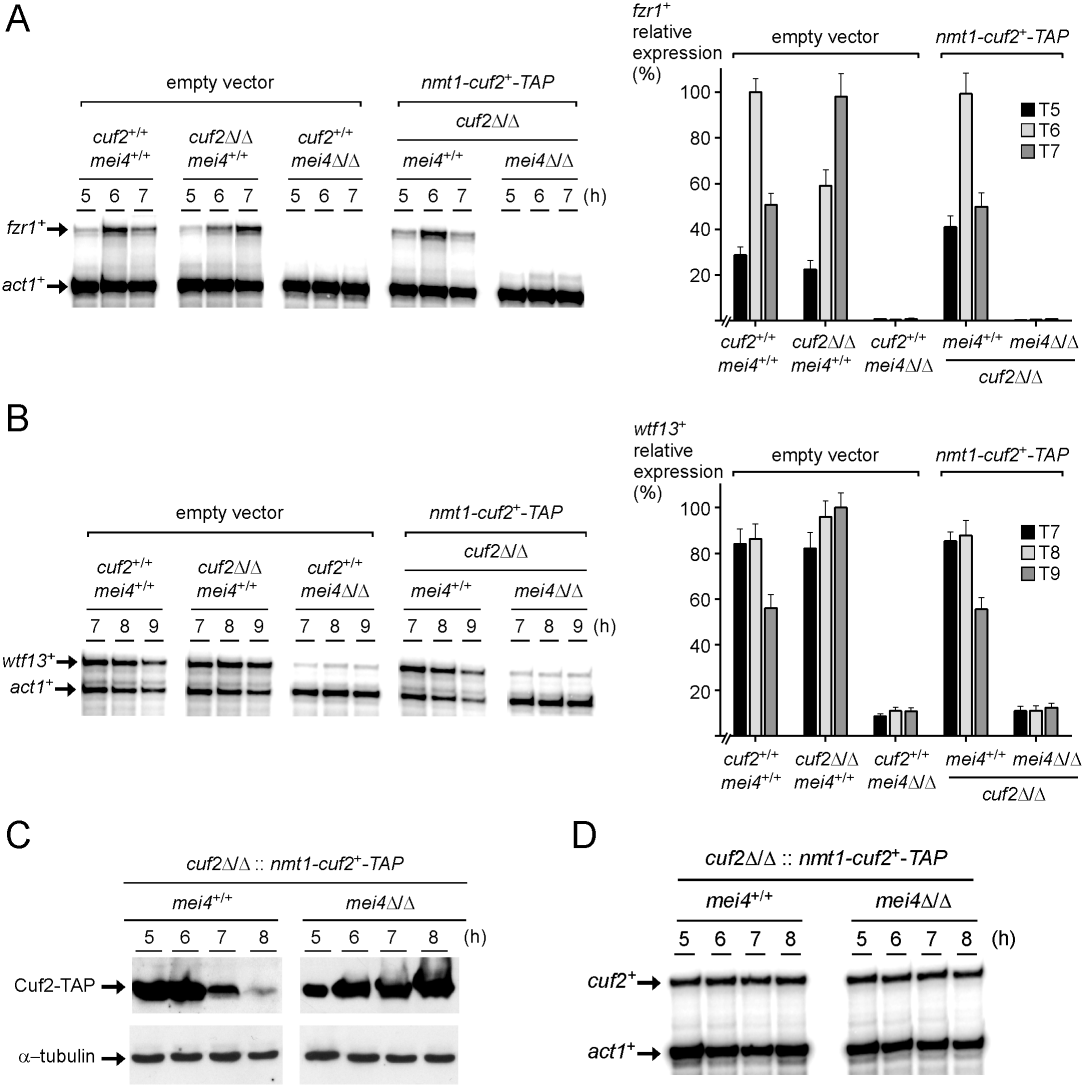
Effect of *mei4Δ/mei4Δ* deletion on the expression of genes that are under the control of Cuf2. A–B, *pat1-114/pat1-114 cuf2*^*+*^*/cuf2*^*+*^, *pat1-114/pat1-114 cuf2Δ/cuf2Δ*, and *pat1-114/pat1-114 cuf2Δ/cuf2Δ mei4Δ/mei4Δ* strains were transformed either with pBPade6 (empty vector) or pBPade6*nmt*^*+*^*cuf2*^*+*^*-TAP* (Cuf2-TAP). Cultures were pre-synchronized by nitrogen starvation and then induced to undergo synchronous meiosis in the absence of thiamine. Following induction of meiosis, total RNA was isolated at the indicated time points. After RNA preparation, *fzr1*^*+*^ (panel A) and *wtf13*^*+*^ (panel B) steady-state mRNA levels were analyzed by RNase protection assays using actin (*act1*^*+*^) as an internal control. Histograms (right side) show quantification of the results of three independent RNase protection assays, including the experiment shown on the left side. C, Culture aliquots of *cuf2Δ/cuf2Δ* and *cuf2Δ/cuf2Δ mei4Δ/mei4Δ* cells expressing Cuf2-TAP were used to prepare whole protein extracts at the indicated time points after meiotic induction. Samples were analyzed by immunoblotting using anti-IgG and anti-α-tubulin antibodies to detect Cuf2-TAP and α-tubulin steady-state protein levels, respectively. D, Aliquots of the cultures described in panel C were analyzed for steady-state levels of *cuf2*^*+*^ and *act1*^*+*^ mRNAs. Total RNA was isolated and transcript levels of *cuf2*^*+*^ and *act1*^*+*^ (indicated with arrows) were examined by RNase protection assays.

In *S*. *pombe*, the meiosis-specific Mei4 transcription factor induces the expression of a vast majority of middle-phase genes [11]. Genome-wide analyses using DNA microarrays have revealed that transcription of *fzr1*^+^ and *wtf13*^+^ genes is markedly reduced in meiotic cells lacking *mei4*^+^ (*mei4Δ*/*mei4*Δ) [11]. An independent study using Northern blot assays has reported that *fzr1*^+^ transcript was undetectable in *mei4*Δ/*mei4*Δ mutant cells [19]. To corroborate these data and to gain a better understanding of the interrelationship between Cuf2 and Mei4, we examined the individual contribution of each transcription factor in the regulation of *fzr1*^+^ and *wtf13*^+^. Steady-state transcript levels of *fzr1*^+^ and *wtf13*^+^ were analyzed 5 to 7 h and 7 to 9 h after meiotic induction, respectively. Assessment of mRNA levels were performed in diploid *pat1-114/pat1-114 cuf2*^+/+^ *mei4*^+/+^, *pat1-114/pat1-114 cuf2Δ/Δ mei4*^+/+^, *pat1-114/pat1-114 cuf2*^+/+^ *mei4Δ/Δ*, and *pat1-114/pat1-114 cuf2Δ/Δ mei4Δ/Δ* cells. In mutant cells lacking Mei4 (*mei4Δ/Δ*), results showed that *fzr1*^+^ transcript was absent whereas *wtf13*^+^ mRNA was barely detectable (Fig 6). Consistent with our previous results, *fzr1*^+^ mRNA expression was decreased (~40%) at the 6-h time point in *cuf2Δ/Δ mei4*^+/+^ mutant cells. Conversely, at the 7-h time point, inactivation of *cuf2*^+^ (*cuf2Δ/Δ*) resulted in an increased (~48%) of *fzr1*^+^ transcript levels in comparison to those seen in control cells. In the case of *wtf13*^+^, *cuf2Δ/Δ mei4*^+/+^ cells showed sustained higher levels of *wtf13*^+^ expression as compared with those observed in wild-type cells, specifically after 8 and 9 h. These results indicated that Cuf2 and Mei4 co-regulated *fzr1*^+^ and *wtf13*^+^ gene expression. Because *cuf2*^+^ transcript is absent in cells lacking Mei4 (*mei4Δ/Δ*) [14], an inducible *nmt*-*cuf2*^+^-*TAP* allele was created in order to test whether Cuf2 could influence target gene expression independently of Mei4. Accordingly, steady-state transcript levels of *fzr1*^+^ and *wtf13*^+^ were analyzed in diploid *pat1-114/pat1-114 cuf2Δ/Δ mei4*^+/+^ and *pat1-114/pat1-114 cuf2Δ/Δ mei4Δ/Δ* cells carrying an integrated *nmt*-*cuf2*^+^-*TAP* allele. Cells expressing *nmt*-*cuf2*^+^-*TAP* were cultured in thiamine-free media for 12 h prior to synchronization of meiosis. The temperature was then shifted to 34°C so as to inactivate Pat1 and allow cells to undergo synchronous meiosis for 5, 6, 7, 8, or 9 h. This procedure was used to ensure that expression of Cuf2-TAP was optimal from the *nmt1* promoter. When expressed in *cuf2Δ/Δ mei4Δ/Δ* cells, Cuf2-TAP protein was totally ineffective to activate *fzr1*^+^ or to regulate *wtf13*^+^ gene expression, even though we detected robust steady-state levels of Cuf2-TAP protein (Fig 6). This lack of regulation was not due to an interference of the TAP tag on Cuf2 protein as *fzr1*^+^ and *wtf13*^+^ gene regulation was rescued in *cuf2Δ/Δ mei4*^+/+^ cells carrying an integrated *nmt*-*cuf2*^+^-*TAP* allele. Furthermore, results showed that *cuf2*^+^ mRNA levels were constantly expressed 5 to 8 h after meiotic induction under the control of the *nmt*^+^ promoter, regardless of the presence of Mei4 (Fig 6). However, we observed that steady-state levels of Cuf2 protein were more stable in the absence of Mei4 (*mei4Δ/Δ*) in comparison to those seen in *mei4*^+/+^ cells (Fig 6). An explanation for this observation cannot be put forward at this time. Taken together, these results suggested that Mei4 is the major activator and that Cuf2 acts rather as a co-regulator of *fzr1*^+^ and *wtf13*^+^ gene expression.

### In vivo binding of Cuf2 to its target gene promoters requires Mei4

**Fig 7:**
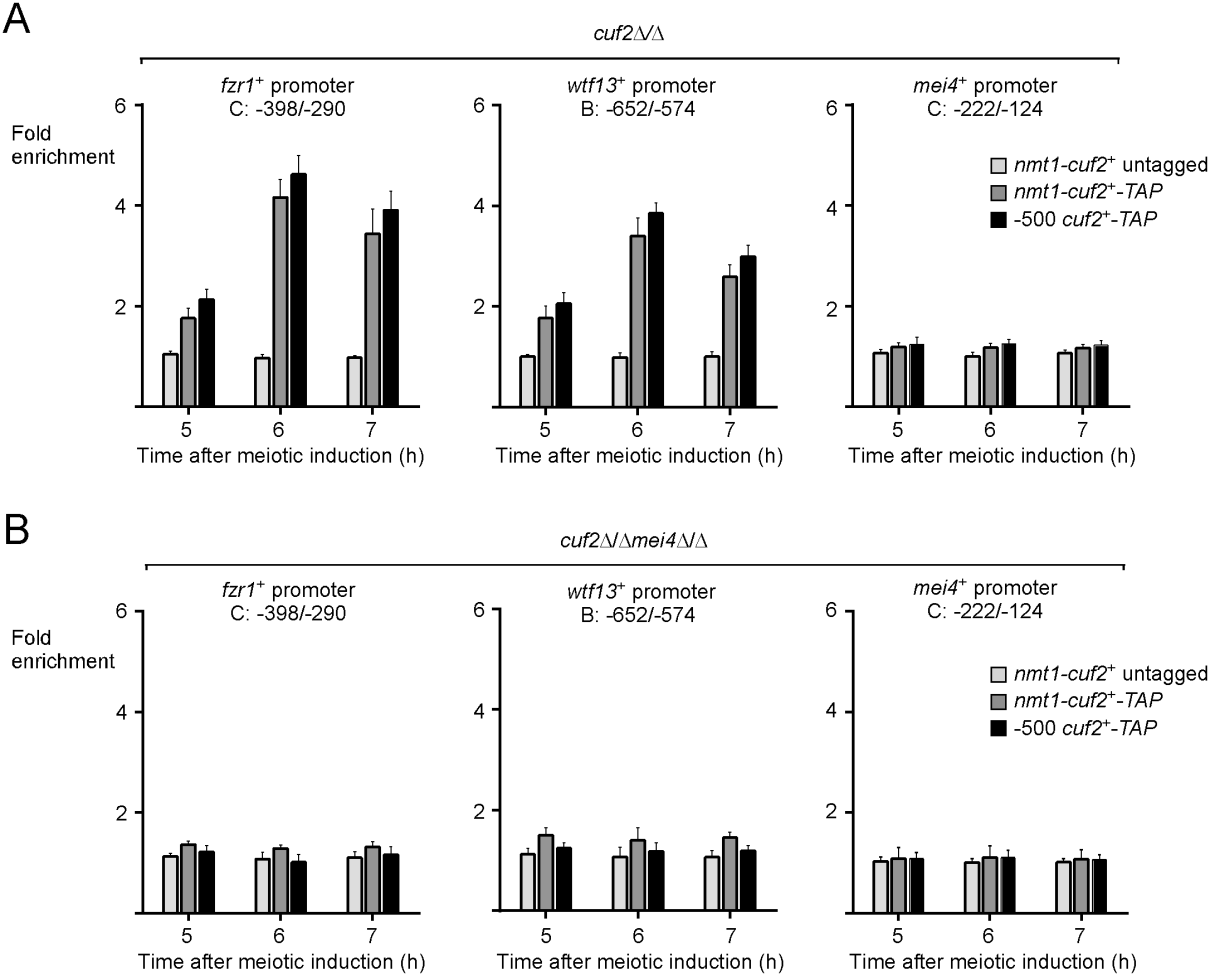
Loss of Mei4 abrogates in vivo chromatin binding of Cuf2. A–B, ChIP analysis of *fzr1*^*+*^, *wtf13*^*+*^, and *mei4*^*+*^ promoters in a *pat1-114/pat1-114 cuf2Δ/cuf2Δ* (panel A) or *pat1-114/pat1-114 cuf2Δ/cuf2Δ mei4Δ/mei4Δ* (panel B) strain expressing an integrated untagged or a TAP-tagged *cuf2*^*+*^ allele under the control of its own promoter or the *nmt*^*+*^ promoter. Chromatin was immunoprecipitated using anti-mouse IgG antibodies. Promoter regions of *fzr1*^*+*^, *wtf13*^*+*^, and *mei4*^*+*^ were analyzed by qPCR to determine Cuf2 chromatin occupancy. TAP-tagged Cuf2 density at *fzr1*^*+*^, *wtf13*^*+*^, and *mei4*^*+*^ promoters was determined as the enrichment of specific *fzr1*^*+*^, *wtf13*^*+*^, and *mei4*^*+*^ promoter regions relative to a 18S ribosomal DNA coding region. Data were calculated as values of the largest amount of chromatin measured (fold enrichment). Results are shown as the averages ± S.D. of a minimum of three independent experiments using distinct chromatin preparations.

Owing to the fact that in the absence of Mei4, Cuf2 by itself was unable to regulate *fzr1*^*+*^ and *wtf13*^*+*^ gene expression, we hypothesized that Mei4 was required to allow the binding of Cuf2 to its target gene promoters. To test this hypothesis, we performed ChIP experiments in diploid *pat1-114/pat1-114 cuf2Δ/Δ mei4*^*+*^*/mei4*^*+*^ and *cuf2Δ/Δ mei4Δ/0394* cells expressing either an untagged or a TAP-tagged version of Cuf2 under the control of the *nmt*^+^ promoter. Enrichment levels of *nmt*^*+*^-induced Cuf2 and Cuf2-TAP proteins were verified at *fzr1*^+^, *wtf13*^+^ and *mei4*^+^ promoters 5 and 7 h after meiotic induction and compared to enrichment levels of Cuf2-TAP expressed from its own promoter used as a reference. At the 6- and 7-h time points, results showed that in *cuf2Δ/Δ mei4*^*+*^*/mei4*^*+*^ cells, *fzr1*^+^ and *wtf13*^+^ promoters were bound by Cuf2-TAP expressed from the *nmt1*^+^ promoter in a manner similar to that observed for Cuf2-TAP expressed from its own promoter (Fig 7). At the 6-h time point, *nmt*^*+*^-Cuf2-TAP and *-500*Cuf2-TAP immunoprecipitated 4.1- and 4.6-fold, respectively, more chromatin corresponding to *fzr1*^+^ promoter compared to a 18S ribosomal region reference (Fig 7). In the case of *wtf13*^+^ promoter, chromatin enrichment levels by *nmt*^*+*^-Cuf2-TAP and -500Cuf2-TAP were 3.4- and 3.8-fold, respectively. Anti-mouse IgG antibodies immunoprecipitated 3.4- and 3.9-fold more Cuf2-TAP (under the control of *nmt*^*+*^ or its own promoter) associated with the *fzr1*^+^ promoter after 7 h of meiotic induction (Fig 7). The same antibodies immunoprecipitated 2.6- and 3.0-fold more Cuf2-TAP associated with *wtf13*^+^ promoter after 7 h. At the 5-h time point, ChIP analysis showed that Cuf2-TAP levels of occupancy were lower at *fzr1*^+^ and *wtf13*^+^ promoters, exhibiting 1.8- and 2.1-fold enrichment. The results from ChIP experiments showed that Cuf2-TAP failed to associate with a *mei4*^*+*^ promoter sequence, which was used as a negative control (Fig 7). In *cuf2Δ/Δ mei4Δ/Δ* cells, Cuf2-TAP (either expressed from *nmt*^*+*^ or its own promoter) did not significantly associate with chromatin that corresponds to *fzr1*^+^, *wtf13*^+^ or *mei4*^*+*^ promoter (Fig 7). Taken together, these results revealed that association of Cuf2 with *fzr1*^+^ and *wtf13*^+^ promoters *in vivo* requires the presence of the transcription factor Mei4.

### Cuf2 and Mei4 mutually interact when they are ectopically expressed in vegetative cells

**Fig 8:**
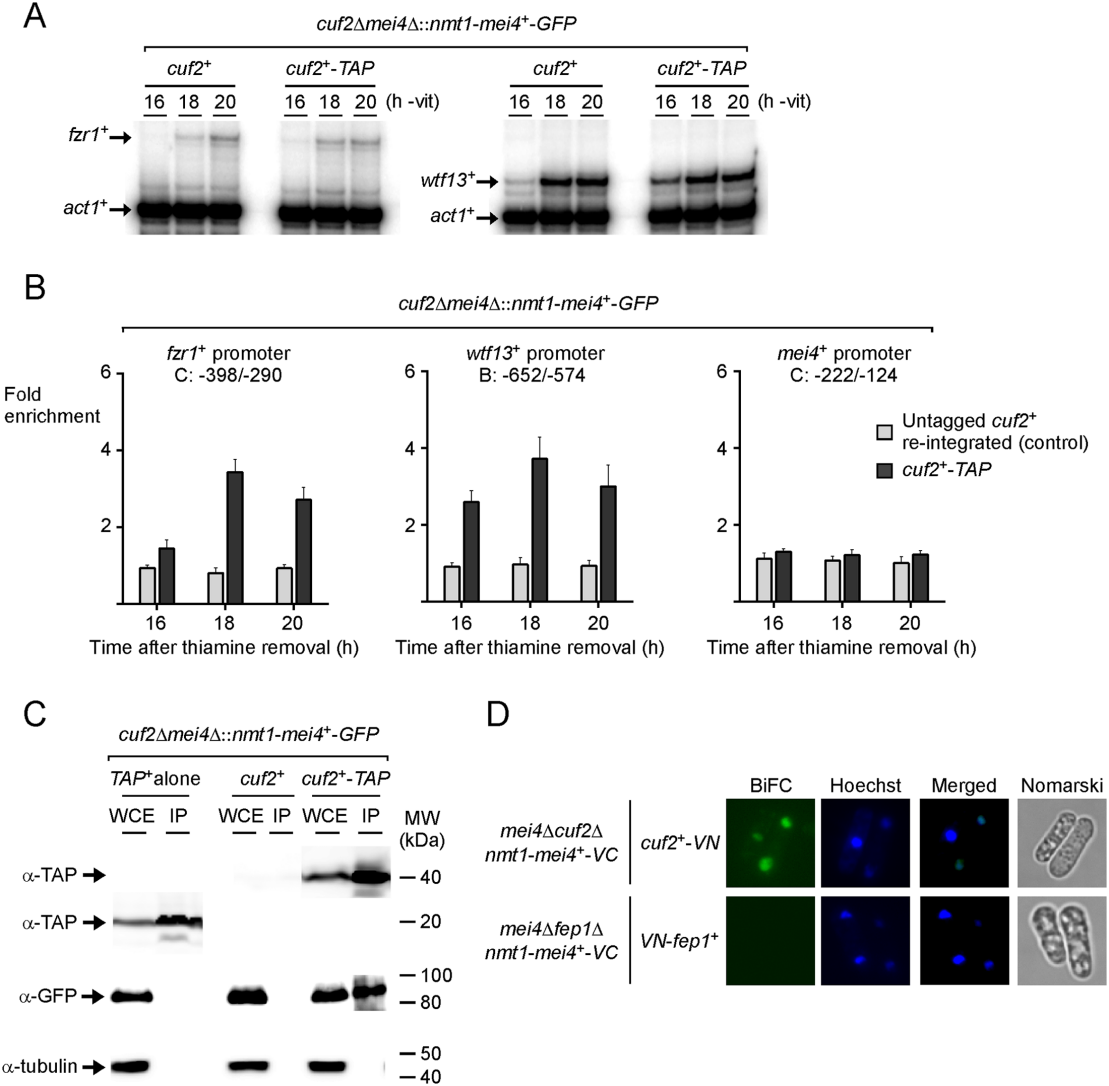
Cuf2 and Mei4 co-expressed in cells proliferating in mitosis interacted with each other. A, *cuf2Δ mei4Δ* cells were co-transformed with untagged or TAP-tagged *cuf2*^*+*^ and *nmt*^*+*^*mei4*^*+*^*-GFP* fusion alleles. When expressed in cells proliferating in mitosis in thiamine-free medium, Mei4 ectopically induced expression of middle-phase meiotic genes, including *fzr1*^*+*^, *wtf13*^*+*^, and *cuf2*^*+*^. Representative expression profiles of the *fzr1*^*+*^ and *wtf13*^*+*^ mRNAs after 16 to 20 h of thiamine starvation are shown. B, Chromatin was immunoprecipitated and promoter regions of *fzr1*^*+*^, *wtf13*^*+*^, and *mei4*^*+*^ were analyzed by qPCR to determine Cuf2 occupancy. TAP-tagged Cuf2 density at *fzr1*^*+*^, *wtf13*^*+*^, and *mei4*^*+*^ promoters was determined as the enrichment of specific *fzr1*^*+*^, *wtf13*^*+*^, and *mei4*^*+*^ promoter regions relative to a 18S ribosomal DNA coding region. Data were calculated as values of the largest amount of chromatin measured (fold enrichment). Results are shown as the averages ± S.D. of a minimum of three independent experiments using separate chromatin preparations. C, Mitotic cells artificially co-expressing GFP-tagged Mei4 and TAP-tagged Cuf2, GFP-tagged Mei4 and untagged Cuf2, or GFP-tagged Mei4 and TAP alone were grown in thiamine-free medium for 18 h. Whole-cell extracts (WCE) were subjected to immunoprecipitation (IP) using IgG-Sepharose beads. The bound proteins were eluted and analyzed by immunoblot assays using a mouse anti-GFP antibody (α-GFP). As controls, aliquots of whole-cell extracts and bound fractions were probed with an anti-mouse IgG antibody (α-IgG) and an anti-tubulin antibody (α-tubulin). D, Cells were grown to early-logarithmic phase and then transferred to a thiamine-free medium for 18 h. Mitotically growing cells co-expressing Mei4-VC and Cuf2-VN or Mei4-VC and VN-Fep1 were visualized by fluorescence microscopy using BiFC (far left) and Hoescht stain (center left). The merged images are shown in the center right panels, whereas Nomarski optics that were used to examine cell morphology are shown in far right panels.

Given the fact that Mei4 was required for binding of Cuf2 to chromatin, protein-protein interaction assays were performed to investigate the possibility that Cuf2 physically associated with Mei4 in *S*. *pombe*. To perform these assays, we chose to artificially express Mei4 in vegetative cells as reported previously [11, 25, 26]. This approach was used because Mei4 is less susceptible to proteolytic degradation in this system and has been shown to functionally induce middle-phase meiotic genes (>306 genes), including *cuf2*^+^, *fzr1*^+^, and *wtf13*^+^. To assess that *fzr1*^+^ and *wtf13*^+^ mRNAs were expressed upon ectopic expression of Mei4 in vegetative cells, we created a *cuf2Δ mei4Δ* double mutant strain in which a functional *nmt*^*+*^*mei4*^*+*^*-GFP* allele was introduced either with an untagged *cuf2*^*+*^ or with a TAP-tagged *cuf2*^*+*^ allele under the control of its own promoter. Mid-logarithmic cells containing the indicated alleles were grown in the absence of thiamine to induce Mei4-GFP expression, which in turn activated ectopic transcription of middle-phase meiotic genes. *fzr1*^+^ and *wtf13*^+^ transcripts were probed 16, 18, and 20 h after induction of the *nmt*^*+*^ promoter. In the case of *fzr1*^+^, results showed that its transcript was detected 18 h after the removal of thiamine from culture media. Subsequently, *fzr1*^+^ mRNA levels displayed a slight increase at later time point (20 h) (Fig 8). In the case of *wtf13*^+^, its expression profile exhibited an earlier response where transcript levels were detected 16 h after the removal of thiamine from culture media. At later time points (18 and 20 h), *wtf13*^+^ mRNA levels were higher in comparison to those observed after 16 h (Fig 8). Regardless of their detection time points and magnitude of expression, *fzr1*^+^ and *wtf13*^+^ mRNAs were induced in a Mei4-dependent manner in vegetative cells, a condition where normally they are expected not to be expressed [14, 19, 20].

To test whether Cuf2 occupied *fzr1*^+^ and *wtf13*^+^ promoter regions under conditions where Mei4 was artificially expressed in vegetative cells, ChIP assays were used to assess the levels of promoter occupancy by a functional Cuf2-TAP after *mei4*^*+*^*-GFP* expression was induced for 16, 18, and 20 h. Results showed that Cuf2-TAP occupied *fzr1*^*+*^ and *wtf13*^*+*^ promoters with 3.4- and 3.7-fold enrichments respectively (relative to a 18S ribosomal DNA coding region used as a reference) 18 h after Mei4-GFP induction (Fig 8). Although the association of Cuf2-TAP with *fzr1*^*+*^ and *wtf13*^*+*^ promoters was weaker 16 h (1.4- and 2.6-fold, respectively) and 20 h (2.7- and 3.0-fold, respectively) after Mei4-GFP induction, presence of Mei4 resulted in an interaction of Cuf2 with these promoters *in vivo*. In contrast, there was no enrichment of Cuf2-TAP detected with the *mei4*^*+*^ promoter, as expected (Fig 8). Ectopic expression of Mei4-GFP in vegetative cells was confirmed by fluorescence microscopy and by observation of membranous structures within cells as reported previously [26].

To further investigate the relationship between Mei4 and Cuf2, TAP pull-down experiments were performed using vegetative cells in which Mei4-GFP was ectopically induced for 18 h. Under these conditions, there is co-expression of *cuf2*^*+*^*-TAP* and most of the middle-phase meiotic genes that include *fzr1*^*+*^ and *wtf13*^*+*^ (Fig 8) [11]. Two additional strains co-expressing either *nmt*^*+*^*mei4*^*+*^*-GFP* and *untagged cuf2*^*+*^ or *nmt*^*+*^*mei4*^*+*^*-GFP* and (unfused) *TAP* alleles were used as controls. Total cell extracts were incubated in the presence of IgG-Sepharose beads that selectively bound (unfused) TAP polypeptide or TAP-tagged Cuf2. In the latter case, it allowed an enrichment of Cuf2 and detection of putative interacting partners. Western blot analysis of the retained proteins revealed that Mei4-GFP was present in the immunoprecipitate fraction when Cuf2-TAP was retained by IgG-Sepharose beads (Fig 8). In contrast, Mei4-GFP was absent in the bound fraction of cells co-expressing TAP alone or untagged Cuf2 (Fig 8). Fractionation of the pull-down experiments was validated using an antibody directed against α-tubulin. Results showed that α-tubulin was present in total cell extracts but not in the retained protein fraction (Fig 8). To assess the steady-state protein levels of Cuf2-TAP, Western blot analyses of both the protein preparations and the bound fractions were performed using anti-IgG antibody (Fig 8).

Given that Cuf2 associated with Mei4 in a protein complex in pull down assays, we investigated their capacity to interact in vivo in *S*. *pombe*. In these experiments, Venus N-terminal fragment (VN) and Venus C-terminal fragment (VC) were fused to the C-terminal portions of Cuf2 and Mei4, respectively. *nmt*^*+*^*mei4*^*+*^*-VC* and *cuf2*^*+*^*-VN* alleles were co-transformed in *mei4Δ cuf2Δ* vegetative cells. After washing media and removing thiamine to express Mei4-VC, which itself activated the allele (*cuf2*^*+*^*-VN*) encoding Cuf2-VN, we examined cells by fluorescence microscopy. After 18 h, the VN-tagged Cuf2 and VC-tagged Mei4 produced BiFC signals, indicating that Cuf2 and Mei4 were forming heteromeric complexes (Fig 8). Cuf2-VN-Mei4-VC fluorescent complexes were seen primarily in nuclei (Fig 8). In this system co-expression levels of Cuf2-VN and Mei4-VC were likely artificially higher than endogenous Mei4 levels since Mei4 was under the control of the *nmt*^*+*^ promoter. Fluorescence was observed in cells co-expressing Cuf2-VN and Mei4-VC fusion proteins but not in cells expressing only one of the fusion proteins. Furthermore, there was an absence of BiFC signal in cells co-expressing two unrelated proteins harboring the N- and C-terminal fragments of Venus, such as VN-Fep1 and Mei4-VC (Fig 8D). Taken together, these results indicated that Cuf2 co-expressed with Mei4 in *cuf2Δ mei4Δ* cells proliferating in mitosis interacted with Mei4 and localized to the nucleus where it occupied target gene promoter regions.

### Association of Cuf2 with the *fzr1*^*+*^ promoter requires FLEX motifs

**Fig 9:**
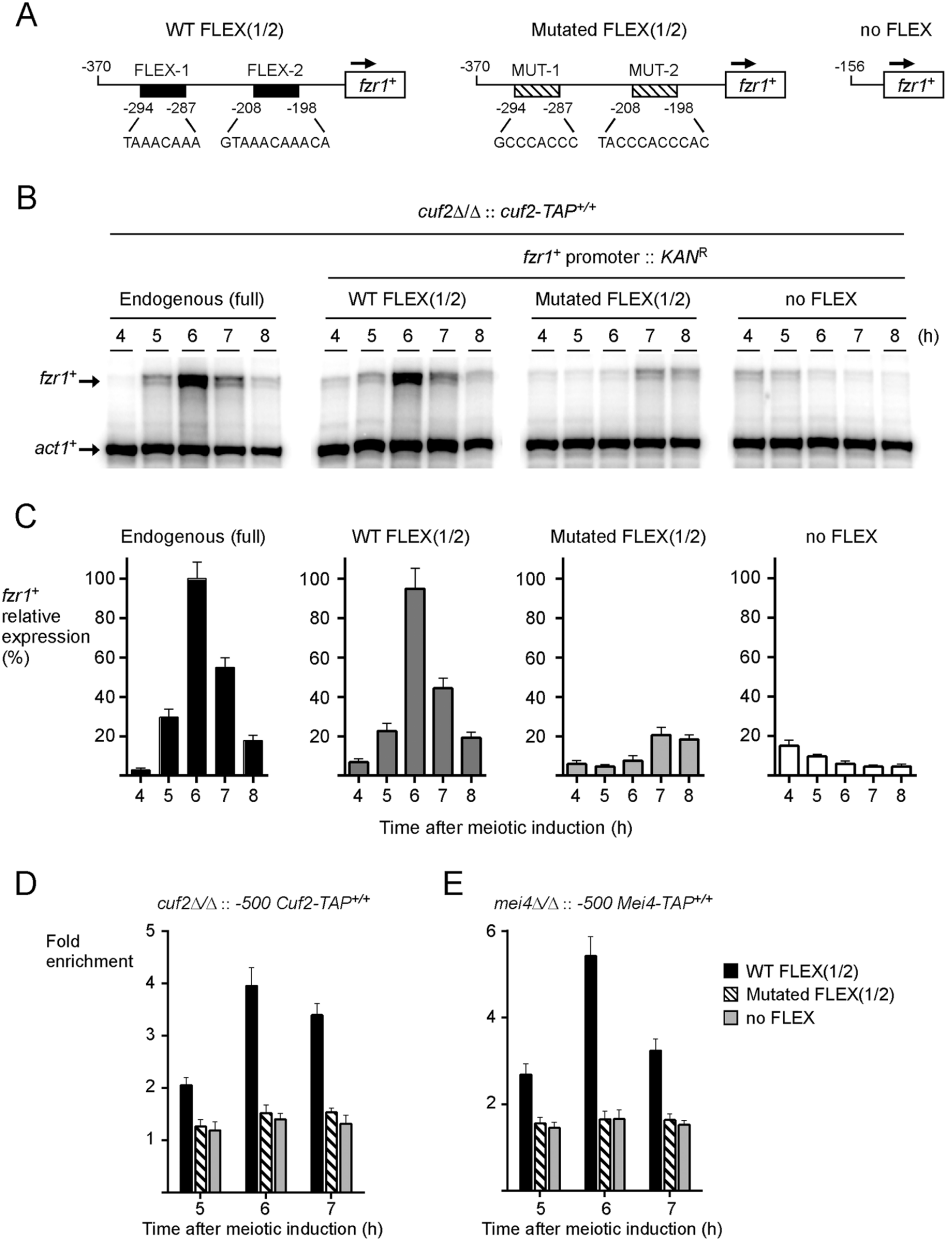
Two FLEX elements in the *fzr1*^*+*^ promoter are required for maximal binding of Cuf2 to chromatin and its ability to co-regulate *fzr1*^*+*^ transcription. A, Schematic representation of a 370-bp *fzr1*^*+*^ promoter DNA fragment and its mutant derivative. An additional 156-bp *fzr1*^*+*^ promoter region without FLEX element is depicted. The black boxes correspond to wild-type FLEX elements, whereas the white boxes represent mutant versions of FLEX. The nucleotide numbers refer to the positions of the cis-acting elements relative to that of the *fzr1*^*+*^ initiator codon. B, Promoter regions shown in panel A were integrated at the chromosomal locus of *fzr1*^*+*^, thereby replacing full-length wild-type promoter by truncated or mutant forms of the *fzr1*^*+*^ promoter. *pat1-114/pat1-114 cuf2Δ/cuf2Δ*::*cuf2*^*+*^*-TAP/cuf2*^*+*^*-TAP* cells containing these modified promoters underwent synchronous meiosis and steady-state levels of *fzr1*^*+*^ and *act1*^*+*^ mRNAs were analyzed at different time points after meiotic induction. C, Quantitative representations of the results of three independent RNase protection assays, including the experiment shown in panel B. Histogram values represent the averages ± SD of triplicate determinations. D, ChIP assays were performed from aliquots of cultures used in panel B. Binding of Cuf2-TAP to the *fzr1*^*+*^ promoter was calculated as the enrichment of the *fzr1*^*+*^ proximal regulatory region relative to a 18S ribosomal DNA coding region. E, ChIP assays were performed to determine Mei4 chromatin occupancy at the *fzr1*^*+*^ promoter using *pat1-114/pat1-114 mei4Δ/mei4Δ*::*mei4*^*+*^*-TAP/mei4*^*+*^*-TAP* cells. Mei4 promoter occupancy was calculated as the enrichment of the *fzr1*^*+*^ proximal regulatory region relative to a 18S ribosomal DNA coding region. For these experiments, full-length wild-type *fzr1*^*+*^ promoter was replaced by modified versions of the *fzr1*^*+*^ promoter shown in panel A.

Mei4 transcription factor contains a highly conserved forkhead-type DNA binding domain that recognizes the (G/A)TAAA(C/T)A consensus sequence known as the FLEX core motif [12, 13]. Given that the presence of Mei4 was required for *fzr1*^*+*^ promoter occupancy by Cuf2, we tested whether binding of Cuf2 to chromatin at the *fzr1*^*+*^ promoter was FLEX-dependent. Using motif-based sequence analysis, we identified two putative FLEX-like elements in the promoter region of the *fzr1*^*+*^ gene (Fig 9). A first element, ^-198^GTAAACAAACA^-208^ (FLEX-1), contained the core heptamer ((G/A)TAAA(C/T)A) and four bases (AACA) in the 3’ flanking sequence that are known to be important for Mei4 binding [12]. A second FLEX-like element (FLEX-2) possessed the sequence ^-287^TAAACAAA^-294^. Although this element lacked the first base of the core heptamer, it contained two ^-293^AA^-294^ in the 3’ flanking sequence that favor Mei4 binding. Furthermore, a similar FLEX element (missing the G-base of the core heptamer) has been found to play an important functional role for transcriptional activation of the meiosis-specific *rem1*^*+*^ gene by Mei4 [40]. To characterize the functional contribution of FLEX-1 and FLEX-2 elements, a shorter version of the *fzr1*^*+*^ promoter (positions -1 to -370) containing wild-type or mutated FLEX elements was integrated at the chromosomal locus of *fzr1*^*+*^, resulting in *fzr1*^*+*^ gene transcription that was under the control of a short promoter region of 370 bp (Fig 9). Results showed that the *fzr1*^*+*^ promoter region containing 370 bp generated an expression profile of *fzr1*^*+*^ mRNA that was virtually identical to that observed in the case of the full-length endogenous *fzr1*^*+*^ promoter (Fig 9). Base pair mutations within the two FLEX elements (TACCCACCCAC instead of GTAAACAAACA, and GCCCACCC instead of TAAACAAA) dramatically reduced the steady-state levels of *fzr1*^*+*^ mRNA 6 h after meiotic induction (Fig 9). Results showed that only low levels of expression were observed, especially 7 and 8 h after meiotic induction. As an additional control, a truncated version of the *fzr1*^*+*^ promoter (positions -1 to -156) lacking FLEX element was also integrated at the chromosomal locus of *fzr1*^*+*^, thereby replacing full-length promoter by a short regulatory region up to -156 from the initiator codon of *fzr1*^*+*^. In this case, *fzr1*^*+*^ transcript levels were very low, exhibiting only a slight increase 4 and 5 h after meiotic induction (Fig 9).

Studies that involved replacement of the full-length *fzr1*^*+*^ promoter by different *fzr1*^*+*^ promoter regions (positions -1 to -370 and positions -1 to -156) at the chromosomal locus of *fzr1*^*+*^ were performed in *cuf2Δ/cuf2Δ* cells expressing Cuf2-TAP or *mei4Δ/mei4Δ* cells expressing Mei4-TAP. We assessed whether wild-type or mutated FLEX elements affected Cuf2-TAP ability to interact with the *fzr1*^*+*^ promoter in vivo. In the presence of wild-type FLEX elements in the context of the promoter region between -370 and -1, results showed that the association of Cuf2-TAP with the *fzr1*^*+*^ promoter was detected with 2.1-, 4.0-, and 3.4-fold enrichment after 5, 6, and 7 h of meiotic induction, respectively, relative to a 18S ribosomal DNA coding region (Fig 9). In contrast, there was no significant Cuf2-TAP chromatin occupancy in cells containing mutated FLEX or in absence of FLEX (positions -156 to -1) (Fig 9). Additional experiments using *mei4Δ/mei4Δ* cells expressing Mei4-TAP showed that wild-type FLEX elements were required for the association of Mei4 with the *fzr1*^*+*^ promoter. Results showed that Mei4-TAP was enriched 2.7-, 5.4-, and 3.2-fold after 5, 6, and 7 h of meiotic induction, respectively. As expected, no significant Mei4-TAP chromatin occupancy was observed in cells containing mutated FLEX or no FLEX (positions -156 to -1) (Fig 9). Taken together, these results showed that multiple point mutations in FLEX elements result in an inability for Cuf2 and Mei4 to interact with the *fzr1*^*+*^ promoter in vivo.

## Discussion

In the present study, we have provided new molecular insights into the mechanism by which Cuf2 regulates the expression of middle-phase meiotic genes. Our data indicates that Cuf2 is a transcriptional co-regulator and has intrinsic antagonistic activities as it can either promote or prevent RNA Pol II occupancy along target gene transcribed regions. When *fzr1*^*+*^ and *wtf13*^*+*^ promoters were analyzed by ChIP assays, results showed that maximal promoter occupancy by Cuf2 occurred 6 h after meiotic induction. In the case of *fzr1*^*+*^, the 6-h time point coincided nicely with maximal chromatin occupancy by RNA Pol II and, consistently, with optimal induction of *fzr1*^*+*^ transcription. In the case of *wtf13*^*+*^ where Cuf2 acts as a negative regulator, data showed puzzling results since *wtf13*^*+*^ mRNA expression was still robust at the 6-h time point, which corresponds to the time point where the association of Cuf2 with the promoter was maximal. Furthermore, results showed a decrease of RNA Pol II occupancy in the presence of Cuf2 at the 6-h time point. The question thus arose how elevated *wtf13*^*+*^ mRNA levels can be explained after 6 h? In *S*. *pombe*, the meiosis-specific *meu5*^*+*^ gene encodes a RNA-binding protein that stabilizes the transcripts of several genes (~188) expressed throughout middle-phase meiosis, including *wtf13*^*+*^ [25]. In the absence of Meu5 (*meu5Δ/Δ*), we have previously reported that *wtf13*^*+*^ mRNA levels were significantly reduced during middle-phase meiosis, as they drastically decreased as soon as 6 h after meiotic induction [14]. In the absence of both Meu5 and Cuf2 (*meu5Δ/Δ cuf2Δ/Δ*), we further observed that *wtf13*^+^ transcript was up-regulated from the 6-h time point, indicating that the premature decrease in the abundance of *wtf13*^+^ transcript in *meu5Δ/Δ* cells was dependent of Cuf2 repressive activity [14]. Therefore, it appears that although Cuf2 reduces RNA Pol II chromatin occupancy at the 6-h time point to repress *wtf13*^+^ transcription, Meu5 simultaneously stabilizes *wtf13*^+^ mRNA to extend its presence at least until 8 h after meiotic induction. In the absence of Cuf2 (*cuf2Δ/Δ*), we have previously shown that *meu5*^*+*^ expression was up-regulated [14]. As a consequence, Meu5 is present for a longer period of time, thereby stabilizing and extending even longer the presence of *wtf13*^*+*^ transcripts to the later time points. These observations suggest that Cuf2 transcriptional control and Meu5 mRNA decay mechanisms act concomitantly to regulate meiotic gene expression.

Cuf2 has been originally identified as a protein sharing a strong sequence homology with an N-terminal 61-residue segment found in metalloregulatory transcription factors involved in either copper transport or detoxification pathways [14, 41–43]. The former pathway includes the *S*. *cerevisiae* Mac1 and *S*. *pombe* Cuf1 transcription factors that are known to activate the expression of genes encoding components involved in high-affinity copper transport [44]. Functional characterization of Mac1 and Cuf1 have revealed that their DNA binding domains are found within their N-terminal regions, corresponding to the first 159 and 174 amino acid residues, respectively [45, 46]. The amino acid sequence similarities between Cuf2 and Cuf1 or Cuf2 and Mac1 are found primarily within the first 60-residue segment of Cuf2, which covers only a fraction of the full-length DNA binding domains of Cuf1 and Mac1. However, similar to Cuf1 and Mac1, Cuf2 harbors a conserved (K/R)GRP motif that may participate in the binding of nucleotides located within the minor groove of the DNA helix [15]. The copper detoxification pathway includes the *S*. *cerevisiae* Ace1 and *C*. *glabrata* Amt1 regulators that become active when yeast cells are grown under high concentrations of extracellular copper ions. Under these conditions, Ace1 and Amt1 activate the expression of metallothionein genes that encode small Cys-rich proteins, which are known to scavenge excess copper, thereby preventing the accumulation of copper to toxic levels. The DNA-binding domain of Ace1 and Amt1 is constituted of two sub-regions. One sub-region encompasses an N-terminal 40-residue segment that contains a (K/R)GRP motif, which shares high homology to the minor-groove-binding domain of the human high mobility group protein HMG-I(Y) [15, 47]. A second sub-region corresponding to amino acid residues 41–110 contains four highly conserved Cys-X_(1/2)_-Cys motifs, which are known to be essential for coordination of four copper ions and high-affinity sequence-specific DNA binding to the major groove [16]. In the case of Cuf2, although its N-terminal region contains a conserved RGRP motif, it lacks two of the four critical Cys-X_(1/2)_-Cys motifs and that makes the formation of the Ace1/Amt1-like copper regulatory domain highly unlikely. Taken together, these comparative observations of metalloregulatory transcription factors suggest that Cuf2 may only contain a partial N-terminal DNA-binding domain and is consistent with the notion that Cuf2 may require an interacting partner for DNA binding efficiency.

Mei4 is a 517-amino acid protein (~58 kDa) that contains a forkhead-type DNA-binding domain within its N-terminus (residues 71 to 182) [13]. This domain of Mei4 binds to FLEX elements containing the heptamer core, (G/A)TAAA(C/T)A, and additional 3’ flanking nucleotides such as AACA, which confer a stronger Mei4-dependent activation response [12]. Mei4 also possesses a transcriptional activation domain that is located in its C-terminal region, corresponding to the last 140 amino acid residues [13]. Since it is well established that Mei4 binds DNA directly through its forkhead domain, the role of the AT-hook minor-groove-DNA-binding motif found in Cuf2 remains ambiguous within the context of a Mei4-Cuf2 hetero-complex formation. Human HMG-I(Y) family transcription factors have been shown to disturb minor-groove chromatin architecture through their AT-hook domain and that in turn enhances the exposition of the adjacent major groove sequence to interacting partners [48]. Sequence analysis has revealed stretches of AT-rich sequences adjacent to the two functional FLEX elements that were identified in *fzr1*^+^ promoter. The bulk of these observations suggest the possibility that Cuf2 makes contact with an AT-rich sequence adjacent to a FLEX motif. If this is the case, subsequent to its interaction with Mei4, Cuf2 could disturb minor-groove architecture and make the FLEX sequence more accessible for Mei4 binding. Given the fact that Mei4 induces the vast majority of middle-phase genes [11] and that the FLEX motif is found in the promoters of almost all of Mei4 target genes [12], we envision that additional Mei4-dependent targets may also be positively regulated by Cuf2 during meiotic divisions.

Three interacting partners of Mei4 had been identified prior to this study. They are U1-70K, Prp11 and Cdc5, which are active components of the spliceosome [40]. It has been shown that the recruitment of these three components at the coding region of *rem1*^*+*^ was Mei4-dependent and that enables RNA splicing from the *rem1*^*+*^ pre-mRNA during middle-phase meiosis [40]. It is unlikely that Cuf2 is involved in the recruitment of these components, at least in the case of *rem1*^*+*^, since we were unable to detect Cuf2 at the *rem1*^*+*^ locus using ChIP assays. The amino acid region of Mei4 that is required for its interaction with U1-70K, Prp11 and Cdc5 has not yet been identified. In the case of Cuf2-Mei4 association, it is likely that the amino acids responsible for their interaction differs from the one involves in the interaction between Mei4 and the components of the spliceosome. Identification of Mei4 and Cuf2 protein regions that are required for their mutual interaction must await a comprehensive deletion mapping analysis of each regulator.

Our results showed that Cuf2 had a positive regulatory effect on *fzr1*^+^ gene transcription and that its association with the promoter of *fzr1*^+^ was FLEX-dependent. In the case of *wtf13*^*+*^, results showed that Cuf2 negatively influenced its expression in a Mei4-dependent manner. Analogous to *fzr1*^*+*^, the promoter region of *wtf13*^*+*^ contains FLEX-like elements (positions -490 to -484, -667 to -661, and -734 to -728) that are located at the proximity of the region (positions -652 to -574) that gave optimal chromatin occupancy by Cuf2. This correlation suggests that FLEXs play a role as *cis*-acting regulatory elements in the Cuf2-dependent transcriptional control of *wtf13*^*+*^. Similarly to *wtf13*^*+*^, microarray analysis has previously shown that the expression of *SPAC1B2*.*03c* and *SPBC947*.*06c* middle-phase meiotic genes was up-regulated, exhibiting a sustained expression that persisted even during late meiosis in cells lacking Cuf2 [14]. We were able to identify two FLEX-like elements located in the promoter of *SPAC1B2*.*03c* (positions -249 to -243 and -46 to -40) and three elements in the promoter of *SPBC947*.*06c* (positions -1442 to -1437, -1322 to -1316, and -1189 to -1195). Interestingly, Cuf2 occupancy of chromatin was maximal using primers at positions -255 to -101 for *SPAC1B2*.*03c* and at positions -1450 to -1301 for *SPBC947*.*06c*, which are located in close proximity with the FLEX-like elements found in these promoters. Together, these observations suggest that Cuf2 may be associated with Mei4 through a FLEX-type element for the timely repression of meiotic genes at the end of middle phase.

The *S*. *cerevisiae* Ndt80 regulator shares functional similarities with Mei4 as they are both primary transcriptional activators of numerous middle-phase meiotic genes [11, 49, 50]. Furthermore, both regulators are required for meiotic cells to exit prophase I, initiate and go through the meiotic divisions. However, Ndt80 and Mei4 differ significantly with respect to amino acid sequences. For instance, Mei4 contains a forkhead-type DNA-binding domain that binds the FLEX motif ((G/A)TAAA(C/T)A), whereas Ndt80 possesses a DNA-binding domain that has an immunoglobulin-like fold, which specifically interacts with MSE elements (NCRCAAAW) [51, 52]. Interestingly, *S*. *cerevisiae* Sum1 transcriptional repressor also binds to these MSE elements and represses the expression of middle-phase meiotic genes during mitosis and both early and late meiosis [53, 54]. When Sum1 and Ndt80 are present at the same time, they compete for binding to MSEs [54]. However, at the onset of middle meiosis, Sum1 is degraded and Ndt80 becomes the unique regulator that binds to MSEs, thereby inducing middle meiotic genes [55]. In *S*. *pombe*, Fkh2 is another member of the forkhead family of transcription factors that has been shown to bind to FLEX motif. Fkh2 has been shown to be involved in mitotic cell cycle control as it represses the expression of several mitotic genes [56, 57]. Interestingly, recent studies have shown that Fkh2 also represses meiotic genes in proliferating cells, including *fzr1*^+^ [58]. Consequently, it is possible that Fkh2 acts as a negative regulator of meiotic middle-phase genes through binding of FLEX motif during phases that precede middle meiosis. If this is the case, Mei4 may need to compete with Fkh2 to activate middle-phase gene expression in a manner similar to the situation that has been characterized for Ndt80 and Sum1 in *S*.*cerevisiae*. In this case, Cuf2 may serve as a co-activator to facilitate Mei4 binding to the FLEX motif, thereby promoting optimal activation of *fzr1*^*+*^ transcription. On the other hand, our data indicates that Cuf2 counteracts the positive effect of Mei4 in the regulation of *wtf13*^+^ gene expression. This negative regulation by Cuf2 may involve an unknown repressive partner that would become active at that stage of meiosis. Although we showed that Cuf2 was recruited to chromatin, we have not been able to identify a specific *cis*-acting DNA regulatory element for Cuf2. Further studies are ongoing to determine how Cuf2 and Mei4 (or other partners) co-regulate gene expression during meiosis.
